# DNA methylation defines regional identity of human intestinal epithelial organoids and undergoes dynamic changes during development

**DOI:** 10.1136/gutjnl-2017-314817

**Published:** 2017-11-15

**Authors:** Judith Kraiczy, Komal M Nayak, Kate J Howell, Alexander Ross, Jessica Forbester, Camilla Salvestrini, Roxana Mustata, Sally Perkins, Amanda Andersson-Rolf, Esther Leenen, Anke Liebert, Ludovic Vallier, Philip C Rosenstiel, Oliver Stegle, Gordon Dougan, Robert Heuschkel, Bon-Kyoung Koo, Matthias Zilbauer

**Affiliations:** 1 Department of Paediatrics, University of Cambridge, Addenbrooke’s Hospital, Cambridge, UK; 2 European Molecular Biology Laboratory, European Bioinformatics Institute, Wellcome Trust Genome Campus, Cambridge, UK; 3 Anne McLaren Laboratory & Department of Surgery, Wellcome Trust-Medical Research Council Stem Cell Institute, University of Cambridge, Cambridge, UK; 4 Wellcome Trust Sanger Institute, Wellcome Trust Genome Campus, Cambridge, UK; 5 Department of Paediatric Gastroenterology, Hepatology and Nutrition, Cambridge University Hospitals, Addenbrooke’s, Cambridge, UK; 6 Wellcome Trust-Medical Research Council Stem Cell Institute, University of Cambridge, Cambridge, UK; 7 Department of Genetics, University of Cambridge, Cambridge, UK; 8 Institute of Clinical Molecular Biology, Christian-Albrechts-University of Kiel, Kiel, Germany

**Keywords:** intestinal epithelium, intestinal stem cell, intestinal gene regulation, intestinal development, intestinal cell lines

## Abstract

**Objective:**

Human intestinal epithelial organoids (IEOs) are increasingly being recognised as a highly promising translational research tool. However, our understanding of their epigenetic molecular characteristics and behaviour in culture remains limited.

**Design:**

We performed genome-wide DNA methylation and transcriptomic profiling of human IEOs derived from paediatric/adult and fetal small and large bowel as well as matching purified human gut epithelium. Furthermore, organoids were subjected to in vitro differentiation and genome editing using CRISPR/Cas9 technology.

**Results:**

We discovered stable epigenetic signatures which define regional differences in gut epithelial function, including induction of segment-specific genes during cellular differentiation. Established DNA methylation profiles were independent of cellular environment since organoids retained their regional DNA methylation over prolonged culture periods. In contrast to paediatric and adult organoids, fetal gut-derived organoids showed distinct dynamic changes of DNA methylation and gene expression in culture, indicative of an in vitro maturation. By applying CRISPR/Cas9 genome editing to fetal organoids, we demonstrate that this process is partly regulated by TET1, an enzyme involved in the DNA demethylation process. Lastly, generating IEOs from a child diagnosed with gastric heterotopia revealed persistent and distinct disease-associated DNA methylation differences, highlighting the use of organoids as disease-specific research models.

**Conclusions:**

Our study demonstrates striking similarities of epigenetic signatures in mucosa-derived IEOs with matching primary epithelium. Moreover, these results suggest that intestinal stem cell-intrinsic DNA methylation patterns establish and maintain regional gut specification and are involved in early epithelial development and disease.

**Significance of this study**

**What is already known on this subject?**

- Human intestinal epithelial organoids (IEOs) are emerging as translational research tool to study epithelial biology in health and disease.
- Early studies described that regional differences in intestinal or colonic IEOs are preserved in culture.

**What are the new findings?**

- Human IEOs from ileum and colon show a stable highly gut segment-specific epigenetic DNA methylation profile that closely reflects their tissue of origin.
- Intrinsically programmed DNA methylation signatures define regional identity of the human intestinal epithelium.
- Human fetal epithelium shows in vitro maturation, indicated by greater epigenetic plasticity and transcriptional changes in culture.
- Human mucosa-derived IEOs are excellent models to investigate DNA methylation dynamics in GI health, development and disease.

**How might it impact on clinical practice in the foreseeable future?**

- Demonstrating stable epigenetic signatures in IEOs that closely match those of primary cells further extends the use of these promising culture models as future translational research tool in disease modelling, drug discovery and regenerative medicine.

## Introduction

The intestinal epithelium is a complex and highly dynamic tissue which performs a variety of functions including digestion and absorption of nutrients, barrier formation and maintenance of intestinal homeostasis. Dividing adult stem cells located at the crypt base give rise to all major epithelial cell lineages and enable complete renewal of the epithelial layer over 3–5 days. The identification of reliable markers and key signalling pathways in adult intestinal stem cells has enabled long-term in vitro propagation of intestinal stem cells within self-organising three-dimensional intestinal epithelial organoids (IEOs).^1 2^ The ability to generate organoids from individual patients provides an unprecedented opportunity to investigate human epithelial cell biology in health and disease. Moreover, organoids from human fetal gut samples can be used to evaluate developmental changes in epithelial cells.^3^

Epigenetic mechanisms are critical regulators of mammalian development, cellular differentiation and tissue-specific functions. One of the main epigenetic mechanisms is DNA methylation of cytosine in the context of CpG dinucleotides. The role of DNA methylation during intestinal development and homeostasis has been studied in freshly isolated human epithelial cells, biopsies and mouse tissues.^4–7^ However, it remains unclear whether DNA methylation signatures in the epithelium are a stable cell-intrinsic property or a process dependent on external cues. Despite the recognised importance of DNA methylation in regulating gene expression and the maintenance of cellular identity, investigations of IEO culture systems have so far mainly relied on gene expression analysis.^1 2 8^ Indeed, some of these studies have demonstrated differences in gene expression in IEOs derived from distinct regions of the gut.^9^ Yet, an underlying functional mechanism explaining how mucosal IEOs retain regional phenotypic differences has not been identified.

Here, we demonstrate the presence of stable, gut segment-specific DNA methylation signatures in human IEOs, which closely reflect those of matching primary purified epithelium and determine regional transcription and cellular function. In contrast, fetal IEOs showed distinct changes in their DNA methylation profile over time towards the paediatric pattern, suggesting the existence of a cell-intrinsic maturation programme that allows development in vitro without the requirement for other cellular components. Lastly, we demonstrate that IEOs from diseased tissue retains altered DNA methylation signatures, highlighting the potential of organoids to model pathophysiology.

## Methods

### Human intestinal samples

Intestinal biopsies were collected from the terminal ileum (TI) and sigmoid colon (SC) from children under 16 years of age as well as adults undergoing diagnostic endoscopy. All patients included had macroscopically and histologically normal mucosa. Fetal intestine was obtained with ethical approval (REC-96/085) and informed consent from elective terminations at 8–12-week gestational age. Fetal gut was dissected and divided into the proximal (small intestine) and distal (large intestine) sections at the ileocaecal junction. Sample details are listed in online supplementary tables S1 and S2.

10.1136/gutjnl-2017-314817.supp1 Supplementary file 1

### Purification of intestinal epithelial cells

Intestinal epithelial cells (IECs) were purified by enzyme digestion and magnetic bead sorting for the epithelial cell adhesion molecule (EpCAM) and purity was assessed as described previously.^7 10^

### Human intestinal epithelial organoid culture

IEOs were cultured according to protocols by Sato *et al* and Fordham *et al*^2 3^ (see online supplementary table S3). Culture of gastric organoids followed a protocol adjusted from Schlaermann *et al*^11^ (see online supplementary table S4).

### RNA and DNA extraction

DNA and RNA were extracted simultaneously from the same sample as described previously.^7^

### Genome-wide DNA methylation arrays

Bisulfite-converted DNA was measured using the Infinium HumanMethylation450 BeadChip (Illumina, Cambridge, UK). The readout for each CpG is expressed as beta value (from 0=unmethylated to 1=fully methylated) or the log2-beta value (M-value). A subset of IEC samples formed part of previous publications.^7 12^

### RNA sequencing

RNA sequencing was performed on the Illumina HiSeq 2500 platform. Further information is provided in the supplement.

### In vitro experiments

IEOs were in vitro differentiated by culturing in differentiation medium (see online supplementary table S5) for 4 days. For Aza-deoxycytidine (AdC) treatment, 1 µM AdC was added for 48 hours followed by a 4-day AdC-free recovery. For long-term AdC treatment, fetal proximal gut -derived IEOs were treated once with 1 µM AdC for 48 hours at passage 1 and then maintained in regular medium for one or five additional passages.

### Bisulfite conversion and pyrosequencing

DNA was bisulfite-converted and locus-specific DNA methylation analysis was performed by pyrosequencing as described previously.^7^ Primer sequences are provided in online supplementary table S6.

### Reverse transcription and quantitative PCR

RNA was reverse-transcribed and used in quantitative PCR (qPCR) as described previously.^7^ Relative expression was calculated using the ΔΔCt method.^13^ Primer sequences are provided in online supplementary table S7.

### Immunofluorescence and imaging

Fluorescent and brightfield images were obtained using an EVOS FL system (Life Technologies). Organoids were stained using anti-FABP6, anti-MUC5B (Atlas Antibodies) or anti-EpCAM (abcam) antibodies. Further details are provided in the supplement.

### Vector construction and genome editing

Plasmid vectors for hCas9 (#41815) and gRNA (#41824) were obtained via Addgene. The targeting vector was generated from human genomic DNA and assembly into pUC118-FLIP-Puro backbone (Addgene #84538).^14^ Further details are provided in the supplement. Human IEOs were electroporated following the protocol from Fujii *et al*.^15^

### Statistical analysis

Statistical analysis for qPCR and pyrosequencing data was performed using GraphPad Prism V.7.00 (GraphPad). Significance levels were determined using multiple t-test with Holm-Sidak correction.

### Bioinformatic analysis

Bioinformatic analysis was performed in R software for statistical analysis V.3.2.3 using Bioconductor V.3.2 packages.

DNA methylation data were analysed using *minfi*^16^, *sva*,^17^ *limma*^18^ and *DMRcate*^19^. *Minfi* was used to generate multidimensional scaling (MDS) plots, which allow visualising similarity between large datasets by reducing complexity to a two-dimensional scale. RNA sequencing data were processed using *fastq_illumina_filter, cutadapt*^20^, *tophat2*,^21^ *bowtie*^22^, *samtools*^23^, *htseq-count*,^24^ *RUVseq*.^25^, *DESeq2*^26^ and *GOseq*.^27^ Further details are provided in the supplement.

## Data availability

Genome-wide data have been deposited in ArrayExpress, accessions E-MTAB-4957 (DNA methylation) and E-MTAB-5015 (RNA sequencing).

## Results

### Paediatric and adult mucosa-derived intestinal organoids retain gut segment-specific DNA methylation profiles

**Figure 1:**
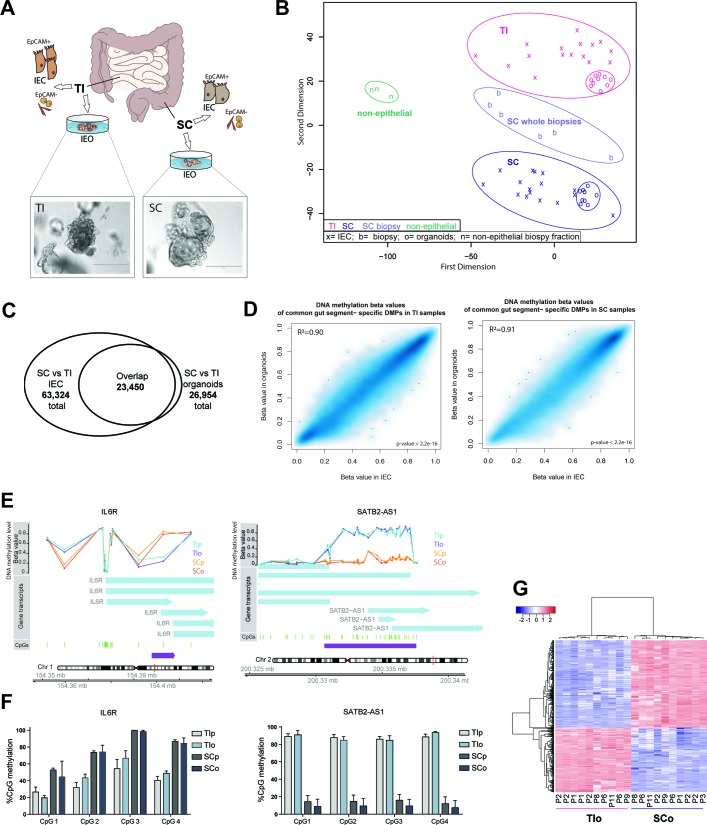
DNA methylation profiling of human intestinal epithelial organoids (IEOs) and primary intestinal epithelial cells (IECs). (A) Summary of experimental setup. Mucosal biopsies were obtained from distal small bowel (terminal ileum=TI) and distal large bowel (sigmoid colon=SC) and used for IEC purification or generation of IEOs. Brightfield images showing established IEOs in culture. (B) Multidimensional scaling plot of genome-wide DNA methylation profiles of purified IEC (labelled as ‘x’), mucosa-derived IEOs (labelled as ‘o’), whole biopsies from SC (labelled as ‘b’) and non-epithelial cell fraction of mucosal biopsies (labelled as ‘n’). (C) Venn diagram indicating the number of significant differentially methylated positions (DMPs) as well as overlap between TI and SC purified primary cells and respective organoids (adjusted p<0.01, n=16 IEC, n=10 IEO for each gut location.) Organoids were passages 1–11. (D) Scatterplots of average DNA methylation beta values of overlapping gut-segment specific DMPs (see figure 1C) in matched IECs and IEOs of the respective gut segment. Pearson correlation r=0.948 (TI) and r=0.955 (SC), p<2.2e-16. (E) Examples of differentially methylated regions displaying gut segment-specific methylation levels in both organoids and primary epithelium. Left panel region upstream of interleukin-6 receptor (*IL6R*), right panel special AT-rich sequence-binding protein 2-antisense 1 (*SATB2-AS1*). Chromosomal location, CpGs and DNA methylation (expressed as average beta value (ranging from 0=unmethylated to 1=fully methylated) of sample groups) are displayed. (F) Validation of DNA methylation profiles by pyrosequencing of bisulfite-converted DNA, showing per cent of CpG methylation in genomic regions of *IL6R* and *SATB2-A*S1. Data are presented as mean+SD of n=4 per group and representative for two independent experiments. (G) Heatmap of top 1000 DMPs, identified by comparing purified TI versus SC epithelium, in the respective organoids profiled at various passages (P). See also online supplementary figures S1 and S2. SCo, SC organoids; SCp, SC purified; TIo, TI organoids; TIp, TI purified.

To investigate DNA methylation and its role in regulating cellular function in human IEOs, we generated organoids from mucosal biopsies from healthy children and adolescents from the distal small bowel (terminal ileum=TI) and distal large bowel (ie, sigmoid colon=SC, figure 1A and online supplementary figure S1A). Additionally, we generated a matching reference sample set of primary, highly purified intestinal epithelium (sorted for the epithelial cell adhesion molecule EpCAM)(figure 1A and refs 7 12). Genome-wide DNA methylation analysis on these samples demonstrated distinct separation of primary epithelium, whole biopsy tissue and non-epithelial, mucosal tissue fraction (figure 1B). Moreover, purified primary IEC displayed highly gut segment-specific DNA methylation profiles as reported previously (figure 1B and refs 7 12). Importantly, IEO cultures clustered closely with primary IEC samples from the same gut segment, indicating that epithelial cells retain a highly gut segment-specific epigenetic profile in culture (figure 1B and online supplementary figure S1B). This was also the case for organoids generated from adult individuals (age 24–60 years, see online supplementary figure S1C and D).

10.1136/gutjnl-2017-314817.supp2 Supplementary file 2

To assess to what extent the DNA methylation profiles of organoids reflect gut segment-specific epigenetic signatures of primary epithelium, we performed differential methylation analysis comparing TI and SC for both organoids and primary epithelial samples (supplementary tables S13 and S14) (supplementary files 3 and 4). The majority (87%) of significantly differentially methylated positions (DMPs) between TI and SC organoids were also differentially methylated in the respective primary purified TI and SC IEC (figure 1C). Strikingly, DNA methylation levels of these gut segment-specific DMPs correlated strongly between IEC and organoids in each segment (figure 1D) and the differences (ie, hypo-methylation/hyper-methylation) occurred in the same direction for almost all (99.8%) overlapping DMPs (see online supplementary figure S2A). Combining adjacent DMPs, we identified several thousand gut segment-specific differentially methylated regions (DMRs) between TI and SC with differences highly preserved in the respective organoids, including interleukin-6 receptor (*IL6R*), special AT-rich sequence-binding protein 2 antisense 1 (*SATB2-AS1*) and claudin 15 (*CLDN15*) (figure 1E, see online supplementary figure S2B). We validated the methylation profiles of selected DMRs with pyrosequencing (figure 1F).

### Gut segment-specific DNA methylation signatures are stable

Organoids derived from the same gut segment and profiled after a range of culture periods (ie, from 1 to 11 passages) displayed close clustering in the MDS plot (figure 1B), indicating that gut segment-specific DNA methylation profiles are highly stable over prolonged culture periods (up to 3 months). Furthermore, no statistically significant DMPs between low and high passage IEOs were identified in any of the gut segments. Additionally, displaying DNA methylation of the top 1000 gut segment-specific CpGs highlighted the clear clustering of samples according to gut segment with no major differences between higher and lower passage organoids (Figure 1G). These findings were confirmed by locus-specific pyrosequencing of early (passage ≤5) and late (passage ≥10) organoids (see online supplementary figure S2C).

### Transcriptional profiles of human IEOs display gut segment-specific signatures of gene expression

**Figure 2:**
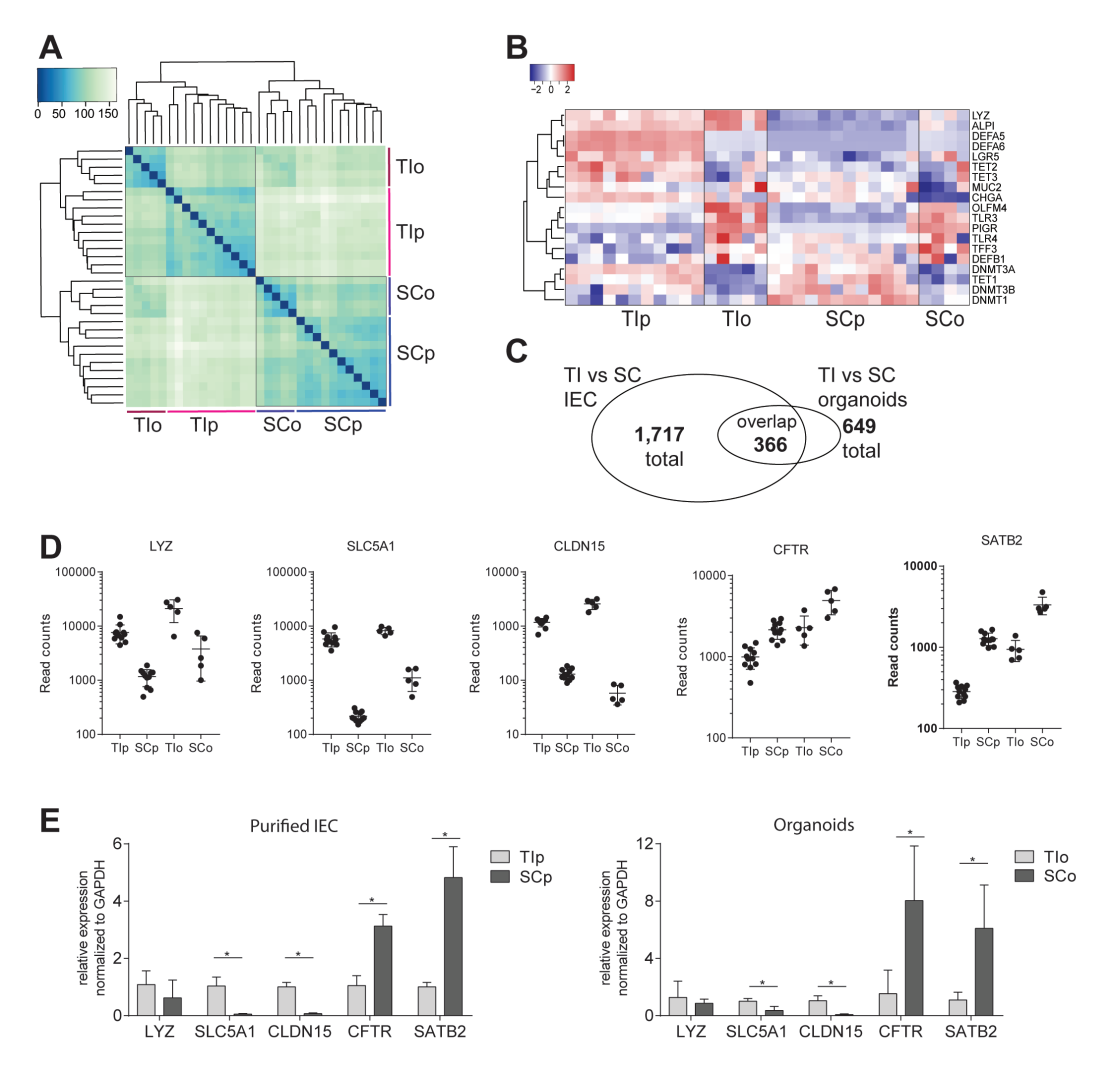
Transcriptomic profiling of human intestinal epithelial organoids (IEOs) and primary intestinal epithelial cells (IECs). (A) Hierarchical clustering and sample heatmap of transcriptomes by RNA-sequencing of IECs and organoids from terminal ileum (TI) and sigmoid colon (SC). (B) Heatmap displaying gene expression (ie, rlog-transformed RNA-seq counts) of selected epithelial cell subset markers, genes involved in intestinal epithelial innate immune function and regulation of DNA methylation. (C) Venn diagram displaying number as well as overlap of differentially expressed genes (DEGs) comparing TI versus SC of primary IEC and organoids. Significance cut-off adj. p<0.01 and log_2_Fold Change>±1.5, n=11 (IEC) and 5 (IEO) for each gut segment. Organoids were passages 1–6. (D) RNA-seq read counts of selected marker genes found to retain gut segment-specific expression patterns in organoid culture. (E) Validation of differentially expressed marker genes by quantitative PCR on a validation sample set, represented as mean+SD, n=3–5 per group, *p<0.05. *ALPI*, intestinal alkaline phosphatase; *CFTR*, cystic fibrosis-transmembrane conductance regulator; *CHGA*, chromogranin A; *CLDN15*, claudin 15; *DEFA*, defensin alpha; *DEFB1*, defensin beta 1; *DNMT*, DNA methyltransferase; *LGR5*, leucin-rich repeat containing G protein-coupled receptor 5; *LYZ*, lysozyme; *MUC2*, mucin 2; *OLFM4*, olfactomedin-4; *PIGR*, polymeric immunoglobulin receptor; *SATB2*, special AT-rich sequence-binding protein 2; *SLC5A1*, solute carrier family 5 member 1; *TET*, ten-eleven translocation; *TFF3*, trefoil factor 3; *TLR*, Toll- like receptor.

In parallel to DNA methylation, we performed transcriptome analysis on organoids and primary IEC by RNA-sequencing (RNA-seq). Unsupervised hierarchical clustering and sample relation analysis of transcriptomes revealed a clear, primary separation of samples according to gut segment (figure 2A). However, within each gut segment organoids were found to separate from primary IEC (figure 2A). The expression levels of several key genes involved in intestinal epithelial cellular function and/or epigenetic regulation are displayed in figure 2B, highlighting both the similarities of samples from the same gut segment and the differences between organoids and primary IEC. Moreover, in contrast to DMPs, only 56% of significantly differentially expressed genes (DEGs) between SC-derived and TI-derived organoids overlapped with DEGs in the respective IEC (figure 2C). However, the majority of overlapping DEGs (98%) showed the same direction when comparing TI and SC. Genes that retain gut segment-specific expression levels in organoid cultures include lysozyme (*LYZ*), the main glucose transporter solute carrier family 5 member 1 (*SLC5A1*), *CLDN15*, cystic fibrosis transmembrane conductance regulator (*CFTR*) as well as *SATB2* (figure 2D). We confirmed expression patterns of some of these key genes identified above by quantitative real-time PCR in an additional sample set (figure 2E; DNA methylation levels are provided in online supplementary figure S3).

### DNA methylation is stable during in vitro differentiation of IEOs and regulates induction of gut segment-specific gene expression

**Figure 3:**
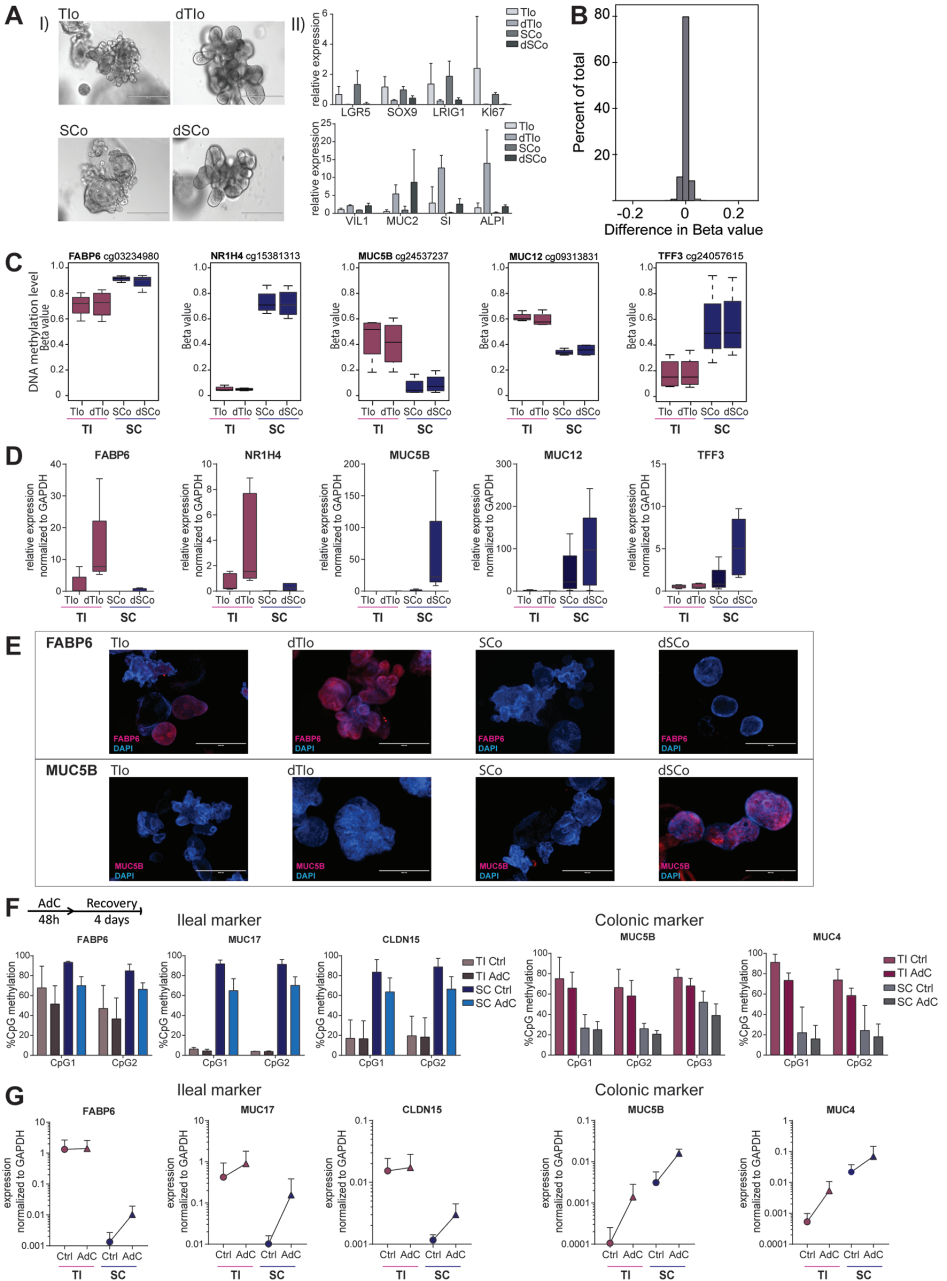
DNA methylation-dependent, gut segment-specific induction of gene expression in intestinal epithelial organoids (IEOs) during in vitro differentiation. (A) (I) Brightfield images of terminal Ileum (TI) and sigmoid colon (SC)-derived IEOs in maintenance medium (TIo and SCo), and differentiation medium (dTIo and dSCo), showing change of gross phenotype and (II) change in intestinal stem cell and epithelial cell subset marker expression measured by real-time PCR. (B) Histogram of mean difference in DNA methylation (expressed as beta values) of all ~450K tested CpGs comparing differentiated with matched undifferentiated intestinal organoids, n=8 derived from four patients (C) DNA methylation level of selected CpGs showing stable gut segment-specific differences in undifferentiated and differentiated IEOs, boxplot of n=4 derived from four patients for each group. Array cg-identifier is listed on top. (D) Real-time PCR data showing induction of gene expression during in vitro differentiation, n=5 per group. (E) Immunofluorescent staining of undifferentiated and differentiated IEOs forFABP6 (red, upper panel) and MUC5B (red, lower panel) protein expression. Blue=DAPI, cell nuclei. (F) CpG methylation quantified by pyrosequencing located within ileal and colonic marker genes in IEOs at baseline (Ctrl) and after co-culture with DNA methyltransferase inhibitor (Aza-deoxycytidine (AdC)) over 48 hours followed by a 4-day recovery period. Data shown as mean+SD of n=4 per group. (G) Gene expression of the markers in (F) shown as absolute values normalised to glyceraldehyde-3-phosphate dehydrogenase (*GAPDH*). Mean+SD, n=3–4 per group. *KI67*, marker of proliferation Ki-67; *LRIG1*, leucine-rich repeats and immunoglobulin-like domains 1; *SI*, sucrase isomaltase; *SOX9*, SRY Box 9; *VIL1*, villin 1.

We hypothesised that the transcriptional differences between organoids and IEC might arise in part from the enrichment for the stem cell niche in maintenance organoid culture. We therefore subjected organoids to in vitro differentiation that mimics migration upwards from the intestinal crypt.^2 8 28^ Differentiated organoids (dIEOs) displayed subtle microscopic differences, along with reduced expression of stem cell markers and increased epithelial subset markers (figure 3A). Genome-wide DNA methylation analysis of differentiated versus undifferentiated IEOs showed that differentiation did not lead to significant DNA methylation changes. The vast majority of CpGs displayed either minimal (i.e. <0.1 change in methylation beta value) or no change (figure 3B), and differential methylation analysis did not yield any significant DMPs.

Next, we investigated the potential impact of gut segment-specific DNA methylation profiles on regulating mRNA expression in dIEOs. We evaluated genes that had shown limited expression differences between segments in undifferentiated IEOs but contained gut segment-specific DMPs and/or DMRs, including fatty acid binding protein 6 (*FABP6*), nuclear receptor subfamily 1 group H member 4 (*NR1H4*), mucins (*MUC5B* and *MUC12*) as well as *TFF3* (figure 3C). Strikingly, dIEOs displayed gut segment-specific gene expression according to the underlying DNA methylation profile (figure 3D). These patterns were observed for several genes, which displayed both small bowel (ie, TI) and large bowel (ie, SC)-specific mRNA induction profiles. We confirmed that changes in gut segment-specific mRNA expression observed on differentiation reflected changes in protein expression by immunostaining of FABP6 and MUC5B (figure 3E). Our findings suggest that stable DNA methylation profiles contribute to regulating gut segment-specific gene expression and the cellular function of human mucosa-derived IEOs on differentiation.

### Disruption of gut segment-specific DNA methylation in IEOs induces aberrant gene expression

To further confirm our hypothesis, we exposed IEOs to the DNA methyltransferase (DNMT) -inhibitor Aza-deoxycytidine (AdC). Treatment with DNMT-inhibitor reduced DNA methylation, primarily at highly methylated loci, and thereby altered gut segment-specific signatures (figure 3F). Importantly, the reduced DNA methylation led to an increase in mRNA expression levels of the respective genes, causing expression of small bowel-specific genes in SC organoids (ie, *FABP6, MUC17, CLDN15*) and large bowel markers in TI organoids (ie, *MUC5B* and *MUC4*) (figure 3G). Hence, DNA methylation is required to maintain gut segment-specific gene expression in IEOs.

### Human fetal IEOs display gut segment-specific DNA methylation which undergoes dynamic changes in culture

**Figure 4:**
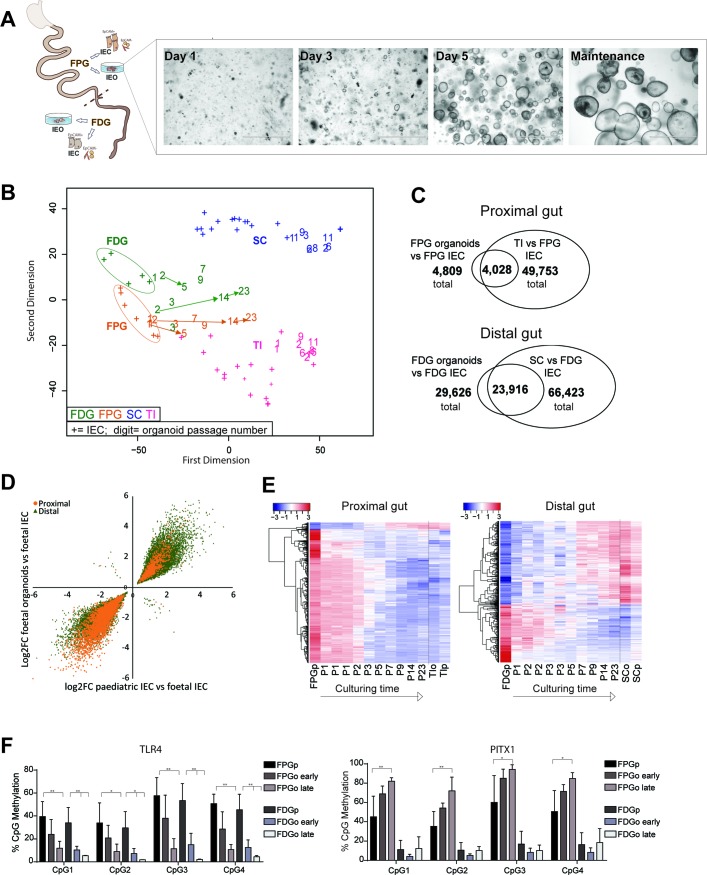
DNA methylation dynamics of human fetal intestinal epithelial organoids (IEOs). (A) Schematic illustration of fetal sample processing and brightfield microscopy images of fetal IEOs at time points 1, 3 and 5 days after seeding and under long-term maintenance conditions. (B) Multidimensional scaling plot of genome-wide DNA methylation profiles of fetal primary intestinal epithelial cells (IECs) and organoids derived from fetal proximal gut (FPG) and fetal distal gut (FDG). Data are displayed in the context of paediatric samples (see figure 1). ‘+’ indicates purified IEC, ‘number’ represents IEO sample and indicates passage. Arrows indicate longitudinal samples of the same IEO line. Fetal organoids: n=10 from eight individuals, paediatric organoids: n=10 from seven individuals (ie, three longitudinal samples). (C) Venn diagrams displaying significant differentially methylated positions (DMPs) (adj. p<0.01) and their overlaps comparing fetal IECs versus fetal IEOs (n=5–10 per group) and fetal IECs versus paediatric IECs (n=5–6 and 16). (D) Correlation plot of fold changes (log2FC) of the overlapping DMPs from (C) (‘maturation-associated DMPs’), indicating that nearly all DMPs show the same direction (ie, higher or lower methylation in fetal IEC with almost 100% concordance for small intestine, 99.7% concordance for large intestine. Correlation: Pearson’s r=0.946 (proximal) and 0.956 (distal), both adj p<0.0001). (E) Heatmap of the top 1000 overlapping DMPs between fetal versus paediatric IECs and fetal IECs versus IEOs (see C) with fetal organoids ordered according to passaging times (P1–P23). As a reference, fetal IECs and paediatric sample values are shown as group average (n=5–16). (F) DNA methylation assessed by pyrosequencing of CpGs located in the promoter region of Toll-like receptor 4 (*TLR4*), left, and paired-like homeodomain 1 (*PITX1*), right, during culture of fetal organoids. Sample groups are fetal IEC, early fetal organoids (passage ≤3) and late fetal organoids (passage ≥10). Mean+SD, n=4–5 per group, *p<0.05 and **p<0.01 versus IEC. See also online supplementary figure S4. FDGo, FDG organoids; FDGp, FDG purified epithelium; FPGo, FPG organoids; FPGp, FPG purified epithelium; SC sigmoid colon; TI, terminal ileum.

In addition to paediatric mucosal biopsies, we established IEO cultures from human fetal proximal gut (FPG, ie, small intestine) and fetal distal gut (FDG, ie, large intestine) (figure 4A and online supplementary figure S4A). Epithelial origin and purity of cultures was confirmed by expression of selected epithelial and non-epithelial marker genes (see online supplementary figure S4B and C). Following generation and long-term culture of these fetal organoids, we compared their genome-wide DNA methylation profiles with a sample set of primary purified human fetal IEC as well as paediatric primary IEC. DNA methylation profiles of primary purified fetal IEC separated distinctly from paediatric samples and displayed some differences according to gut segment (figure 4B and ref.^7^). Similar to paediatric and adult organoids, early passages of fetal organoids clustered close to the respective purified epithelial fraction (figure 4B). Differential DNA methylation analysis indicated that fetal organoids retain a large proportion of gut segment-specific profiles (see online supplementary figure S4D). However, fetal IEOs were found to undergo dynamic DNA methylation changes in culture as higher passage fetal IEOs appeared to cluster closer to paediatric epithelial samples (figure 4B). Given this finding, we aimed to assess whether DNA methylation changes occurring in fetal organoid cultures could indicate a degree of in vitro maturation. We therefore performed differential methylation analysis comparing fetal organoids with primary fetal IEC. We tested for an overlap of identified DMPs with the methylation changes occurring during physiological development from fetal to paediatric epithelium, that is, DMPs between fetal and paediatric primary IEC. Strikingly, the vast majority of identified significant DMPs both for FPG (ie, 83%) and FDG (ie, 80%) indeed overlapped (figure 4C and supplementary tables S15 and S16). Moreover, the direction of DNA methylation changes was the same and correlated strongly in almost all (>99%) overlapping DMPs (figure 4D).

To further assess whether DNA methylation differences between higher passage fetal organoids and fetal IEC truly reflect in vitro maturation of fetal organoids over time, we plotted a heatmap of the DNA methylation levels with increasing passage number (figure 4E) for the top 1000 overlapping DMPs that displayed the greatest difference between fetal and paediatric IEC (from figure 4C). While early passage fetal IEOs displayed DNA methylation levels similar to the matching purified fetal IEC, methylation profiles of higher passage organoids showed high similarities with the respective paediatric IEOs and IECs. These maturation patterns occurred in both directions (ie, gain and loss of methylation) and were present in both fetal proximal and distal organoids (figure 4E). Locus-specific pyrosequencing on an additional sample set confirmed DNA methylation of genes we had identified to be differentially methylated in fetal versus paediatric primary IEC, including the pattern recognition receptor Toll-like receptor 4 (*TLR4*) and the transcription factor paired-like homeodomain 1 (*PITX1*). These genes displayed dynamic DNA methylation and gene expression changes in fetal organoids during prolonged culture (figure 4F and online supplementary figure S4E).

### Dynamic transcriptional changes in human fetal organoids support in vitro maturation

**Figure 5:**
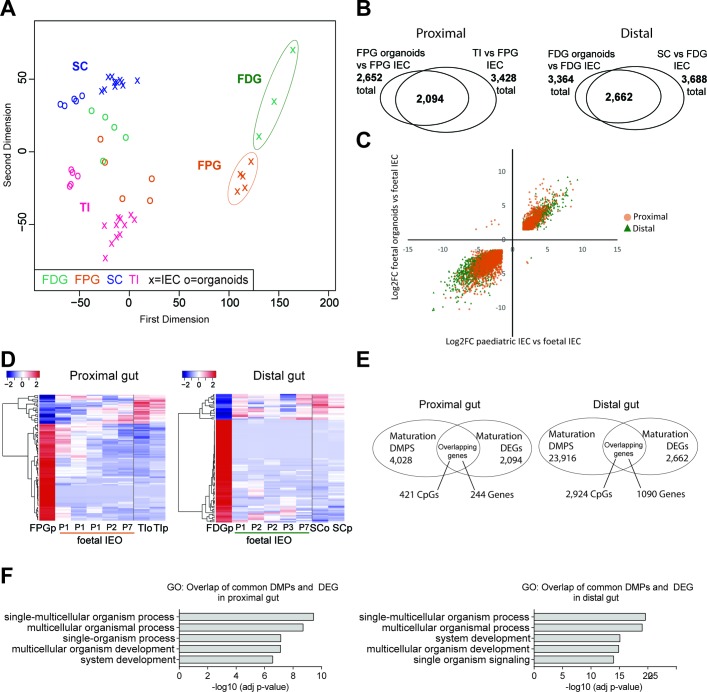
Transcriptional profiling of human fetal intestinal epithelial organoids (IEOs) and correlation with DNA methylation. (A) Multidimensional scaling plot of transcriptome data from fetal intestinal epithelial cells (IECs) and fetal organoids in the context of paediatric primary IECs. (B) Overlap of differentially expressed genes (DEGs) between fetal IEC versus fetal IEOs, and fetal IEC versus paediatric IEC (n=3–5 per fetal group, n=11 per paediatric group), separated for small intestine (left) and large intestine (right). DEG cut-off adj p<0.01 and log2FC>±1.5. (C) Scatterplots of log2FC values for each overlapping DEG transcript from the comparisons in (B). Pearson’s correlation r=0.94 (proximal) and r=0.96 (distal), both p<0.001. (D) Heatmap of top 1000 DEGs previously identified between fetal and paediatric IEC, shown in fetal organoids. Passage number indicated as ‘P’. (E) Venn diagram of overlapping genes that are both differentially methylated and expressed. (F) Five most significant gene ontologies (biological process) for genes both differentially methylated and differentially expressed in vivo and in vitro (see figures 4C and 5B), analysed individually for proximal and distal gut. See also online supplementary figure S5. DMPS, differentially methylated positions; FDG, fetal distal gut; FPG, fetal proximal gut; SC, sigmoid colon; TI, terminal ileum.

To assess the transcriptional dynamics in human fetal organoids, we performed MDS analysis on RNA-seq profiles, which revealed close clustering with paediatric epithelium from the same gut segment (figure 5A). Similarly to DNA methylation, differential gene expression analysis comparing fetal IEC with fetal organoids demonstrated that the majority of identified DEGs overlap with genes differentially expressed between fetal and paediatric IEC, with almost all expression changes (i.e. >99%) occurring in the same direction (figure 5B, C). In support of our hypothesis that fetal organoids undergo in vitro maturation, a heatmap of the top 100 overlapping DEGs (see also figure 5B) further illustrates distinct transcriptional differences between early and late passage fetal organoids with transcriptional profiles appearing more similar to paediatric epithelium (figure 5D).

Assuming a functional association between DNA methylation and gene expression dynamics, we went on to identify genes which displayed both dynamic DNA methylation and gene expression changes in human fetal organoids. In total, we identified 244 and 1090 genes in fetal proximal and distal IEOs, respectively (figure 5E as well as see online supplementary figure S5). Importantly, gene ontology analysis on these genes revealed highly significant enrichments for pathways involved in ‘system development’ and ‘multicellular organism development’ (figure 5F).

### TET1 is involved in regulating in vitro maturation of fetal IEOs

**Figure 6:**
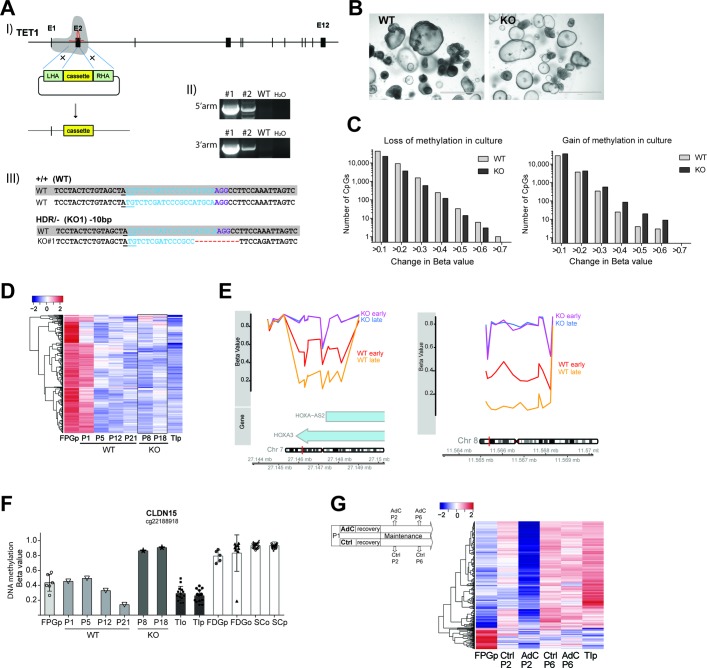
Involvement of ten-eleven translocation 1 (TET1) and DNA methyltransferases (DNMTs) in observed in vitro maturation of human fetal intestinal epithelial organoids (IEOs). (A) Gene targeting of *TET1* in fetal IEOs: (I) Schematic display of targeting strategy using CRISPR-Cas9 and targeting vector, (II) confirmation of homology-directed repair (HDR) by PCR. Knockout (KO) line 1 (#1) was used for subsequent analysis. (III) Sequencing results of *TET1* allele in WT and KO#1 samples aligned to reference. Underline indicates start codon; blue colour indicates gRNA target sequence, purple indicates PAM site. (B) Brightfield images of unaltered fetal proximal gut organoids (FPGo), that is, wildtype (WT) and *TET1-* KO fetal small intestinal IEOs derived from the same individual. (C) Quantification of DNA methylation change between two culturing time points in WT and *TET1*- KO. Plotted are the number of CpG positions displaying either loss (left) or gain (right) of DNA methylation in KO compared with WT organoids during culture. Time points analysed were WT P12 and P21, KO P8 and P18. p=0.02 (loss) and p=0.03 (gain), paired Wilcoxon test. (D) Heatmap displaying DNA methylation of top 1000 maturation-associated differentially methylated positions (see figure 4C) that lose DNA methylation, shown in the respective organoid. (E) Example of differentially methylated regions (DMRs) in the genes for the transcription factors homeobox A3 (*HOXA3*, left) and GATA binding protein 4 (*GATA4,* right) between WT and KO at two different time points in culture. KO early=P8, KO late=P18, WT early=P12, WT late=P21. (F) Beta values of CpG methylation in *CLDN15* at different passages shown in the context of other sample groups. Each symbol represents a sample, bar indicates the average. (G) Effect on dynamic DNA methylation changes in response to treatment with DNMT inhibitors. Heatmap displaying DNA methylation of top 1000 maturation-associated DMPs that gain methylation between fetal and paediatric small intestinal IEC, shown in FPGo at different passages after treatment with Aza-deoxycytidine (AdC). FPG purified epithelium (FPGp) shown as average of n=6, terminal ileum purified (TIp) shown as average of n=16, FPGo shown as average of n=2 per condition. See also online supplementary figures S6 and S7.

Next, we aimed to identify the mechanisms underlying the dynamic changes in DNA methylation suggestive of in vitro maturation of fetal IEOs. Ten eleven translocation 1 (*TET1*) encodes an enzyme that plays a key role in active demethylation of DNA^29^ and is highly expressed in human fetal intestinal epithelium.^7^ To test whether TET1 contributes to the dynamic DNA methylation of fetal IEOs, we applied CRISPR-Cas9 genome editing to human fetal IEOs and generated a TET1-knock-out (KO) fetal proximal organoid line (figure 6A and online supplementary figure S6). Phenotypically, TET1-deficient fetal organoids appeared viable and did not display any obvious differences compared with wildtype (WT) cultures during early passages (figure 6B). However, over time, TET1 KO IEOs showed reduced viability and did not survive beyond 6 months of in vitro culture (online supplementary figure S6B). In addition to these phenotypic differences, a comparison of DNA methylation changes over time (ie, 10 weeks) between KO and WT organoids demonstrated a reduced loss of methylation as well as an increased gain of methylation in the KO fetal organoids (figure 6C). Similarly, methylation of the top 1000 maturation-associated CpGs that lose methylation (see also Figure 4C and D) revealed a reduced loss but rather a gain of DNA methylation between two passaging time points of Tet1-KO organoids (figure 6D). Example CpGs that failed to undergo demethylation in TET1-KO IEOs are shown in figure 6E, F.

In contrast to TET enzymes, DNMTs are involved in maintenance as well as active DNA methylation of the mammalian genome. We therefore expected them to also be involved in regulating in vitro maturation. Accordingly, exposing human fetal organoids to DNMT inhibitors led to global demethylation (figure 6G). Despite this initial loss of DNA methylation, their profile at a later stage revealed a pattern similar to untreated organoids at a similar passage, indicating a transient, reversible effect (figure 6G and supplementary figure S7).

### Human IEOs derived from ectopic gastric mucosa retain altered DNA methylation profiles

**Figure 7:**
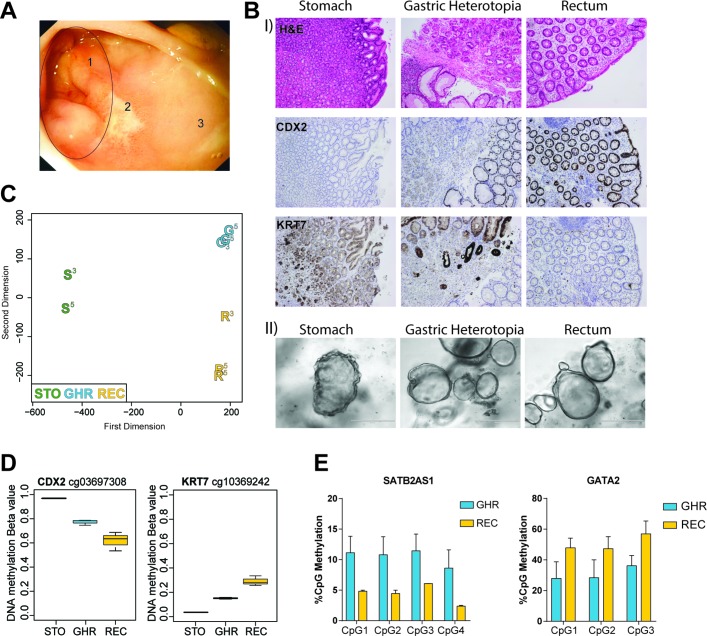
Altered DNA methylation in organoids derived from gastric heterotopia. (A) Endoscopic image of rectal mucosa. Numbers indicate gastric heterotopic region (=1), mucosal ulcer (=2) and healthy, unaffected rectal mucosa (=3). (B) (I) Sections of healthy gastric tissue (STO), gastric heterotopic tissue in the rectum (GHR) and adjacent healthy rectum (REC). Shown are H&E staining as well as immunohistochemistry staining for hindgut marker CDX2 and gastric marker KRT7. (II) Intestinal epithelial organoids (IEOs) derived from stomach, gastric heterotopic and healthy rectal tissue. (C) Multidimensional scaling plot of genome-wide DNA methylation of different IEOs from the same donor. Samples were passages 3 and 5, indicated by superscript number next to the sample. STO-organoids were grown in gastric medium, REC-organoids in intestinal medium and GHR-organoids in both gastric (P3 and P5) and intestinal medium (P5). (D) Methylation levels of CpGs located in the genes of *CDX2* (left) and *KRT7* (right). Box plot of n=2–3 IEO per group. (E) Pyrosequencing data showing percentage of methylation in the genes of *SATB2-AS1* and *GATA2* in heterotopic and rectal IEOs. Data shown as mean+SD of n=2 per group. See also online supplementary figure S8. G, gastric heterotopic organoids; R, rectal organoids; S, stomach-derived organoids.

As demonstrated above, human IEOs derived from healthy intestinal mucosa retain a highly gut segment-specific DNA methylation profile in long-term culture despite significant environmental changes. We therefore wanted to investigate whether IEOs derived from diseased intestinal mucosa also retained a specific DNA methylation profile. We clinically diagnosed gastric heterotopia in the rectum (GHR) of a paediatric patient (figure 7A). The presence of heterotopic gastric mucosa was confirmed using immunostaining for the hindgut marker caudal type homeobox 2 (CDX2) and the gastric marker keratin 7 (KRT7). Mixed staining for both gastric (ie, KRT7) and intestinal (ie, CDX2) markers in heterotopic tissue indicated the presence of a mixed epithelial cell population (figure 7B).

Next, we generated IEOs from gastric, rectal and gastric heterotopic biopsy samples (figure 7B) and subjected them to genome-wide DNA methylation profiling. Unsupervised analysis revealed distinct methylation differences between organoids derived from heterotopic tissue and adjacent, normal rectal mucosa (figure 7C). Interestingly, methylation profiles of gastric heterotopic organoids also differed substantially from gastric organoids, indicating the presence of disease-specific alterations. Importantly, similar to healthy IEOs, DNA methylation profiles of gastric heterotopia-derived IEOs remained overall stable (ie, passages 3–5) independent of gastric or colonic culture conditions (see ‘Methods’) (figure 7C). Among the genes harbouring stable, differentially methylated CpGs were *CDX2* and *KRT7* (figure 7D) and the gene locus around GATA-binding protein 2 (*GATA2*) (see online supplementary figure S8A,B). Pyrosequencing of additional loci (SATB2-AS1 and GATA2) confirmed the distinct DNA methylation differences between heterotopic organoids and healthy rectal mucosa-derived organoids which were also found to lead to subtle changes in gene expression in the case of GATA2 (figure 7E and see online supplementary figure S8C).

## Discussion

IEO cultures are rapidly emerging as powerful models to study intestinal epithelial cell biology. Studies on both human and murine organoids have demonstrated that organoids can retain regional functional properties even in heterotopic grafts.^9 30^ However, the underlying mechanisms remain to be defined.

We have previously shown that DNA methylation profiles of the epithelium in the small and large bowel differ substantially.^7 12^ Our current results using organoid culture indicate that these differences originate in the respective intestinal stem cell population and do not represent epithelial cell subset differences on differentiation. We demonstrate for the first time that IEO cultures retain a highly gut segment-specific DNA methylation profile that reflects that of primary IEC, despite being removed from key environmental factors such as signalling from the underlying mucosa. Hence, our study suggests that these epigenetic signatures represent a highly stable, epithelial-cell intrinsic programme. These findings not only provide further validation for the reliability of human, mucosa-derived IEOs as an important in vitro model system, but also provide novel insight into the critical role of stable DNA methylation in keeping the memory of epithelial tissue origin over a prolonged in vitro culture.

In addition to DNA methylation, we confirmed that transcriptional profiles of paediatric mucosa-derived organoids displayed major similarities with primary epithelium derived from the respective gut segment.^2^ However, we also observed significant differences in the gene expression profiles of organoids compared with primary epithelium. These differences may be caused by a number of factors, including the lack of exposure to several environmental stimuli such as the underlying mucosa, food antigens and adjacent gut microbiota. Additionally, limited cellular differentiation into epithelial subsets under maintenance culture conditions is likely to contribute to a reduced number of regional DEGs in organoids. In line with this, in vitro differentiation led to the expected major changes in gene expression but no changes to the DNA methylation profile. This is in keeping with mouse data that demonstrated minimal differences in DNA methylation between crypt and villus epithelium.^4 31^ Most importantly, we were able to demonstrate that the level of transcriptional changes during cellular differentiation was associated with the underlying, gut segment-specific DNA methylation profile. These findings suggest that stable DNA methylation profiles in mucosa-derived human IEOs play a key role in regulating cellular function in a gut segment-specific manner.

The use of immature fetal epithelium represents another promising area within the field of IEOs.^3 32–34^ We further expand on these early reports by demonstrating successful generation and long-term culture of IEOs derived from human fetal proximal and distal gut. Moreover, in contrast to paediatric and adult IEOs, human fetal IEOs undergo substantial, dynamic DNA methylation as well as transcriptional changes in culture. These striking observations highlight distinct differences in the epigenetic plasticity during various stages of physiological development. Indeed, a number of studies performed in mice have demonstrated substantial changes in DNA methylation of IEC both during embryogenesis and during the early postnatal phase.^4 5^ Interestingly, the vast majority of epigenetic and transcriptional changes observed in fetal organoids during long-term culture were found to overlap with those that differ between purified fetal and paediatric epithelial cells. Together, these findings indicate that human fetal IEOs undergo a degree of in vitro maturation and hence could serve as useful models to investigate epithelial development. Furthermore, we show that genome editing can be used to elucidate the molecular mechanisms of cellular maturation during development.

In addition to modelling human intestinal epithelial biology in health, IEOs also hold great promise for studying diseases. A number of reports have demonstrated that patient-derived organoids display distinct disease-associated phenotypes.^35 36^ Here, we demonstrate that human IEOs from diseased, ectopic mucosal samples retain an altered DNA methylation signature. In light of the observed stability of DNA methylation signatures in organoid cultures and its value as a marker for cellular identity, our data highlight the great potential of organoids to serve as translational research tools in studying the role of DNA methylation in gut diseases.

In summary, our study demonstrates the critical importance of precise intestinal epithelial cell-intrinsic epigenetic regulation in defining regional gut specification during development, in homeostasis and disease pathogenesis.

10.1136/gutjnl-2017-314817.supp3 Supplementary file 4

10.1136/gutjnl-2017-314817.supp4 Supplementary file 3

10.1136/gutjnl-2017-314817.supp5 Supplementary file 5

10.1136/gutjnl-2017-314817.supp6 Supplementary file 6
