# Supplementary material for: DNA methylation defines regional identity of human intestinal epithelial organoids and undergoes dynamic changes during development
Source: Gut. 2017 Nov 15;68(1):49–61. doi: 10.1136/gutjnl-2017-314817 (PMC6839835; doi:10.1136/gutjnl-2017-314817)
Supplement: Supplementary file 1 [file gutjnl-2017-314817supp001.pdf]

Supplementary Material and Methods

Table S1. Patient information for the samples used for DNA methylation arrays

Sample ID	Age group	Sex	Age	Sample Type	Gut Location	Passage	Treatments	Ref
1	paediatric	M	13 yrs	purified	TI and SC	na		[1,2]
7	paediatric	F	14 yrs	purified	TI and SC	na		[1,2]
12	paediatric	F	15 yrs	purified	TI and SC	na		[2]
15	paediatric	F	15 yrs	purified	TI and SC	na		[2]
22	paediatric	F	14 yrs	purified	TI and SC	na		[2]
23	paediatric	M	14 yrs	purified	TI and SC	na		[1,2]
31	paediatric	M	9 yrs	purified	TI and SC	na		[2]
39	paediatric	M	14 yrs	purified	TI and SC	na		[1,2]
42	paediatric	M	15 yrs	purified	TI and SC	na		[2]
56	paediatric	F	13 yrs	purified	TI and SC	na		[1,2]
63	paediatric	M	6 yrs	purified	TI and SC	na		[2]
64	paediatric	M	13 yrs	purified	TI and SC	na		[2]
71	paediatric	M	9 yrs	purified	TI and SC	na		[2]
100	paediatric	M	14 yrs	purified	TI and SC	na		[2]
P1	paediatric	M	13 yrs	purified	TI and SC	na		[1]
P2	paediatric	F	8 yrs	purified	TI and SC	na		[1]
200	paediatric	M	12 yrs	organoids	TI and SC	6		
212	paediatric	M	11 yrs	organoids	TI and SC	6		
212	paediatric	M	11 yrs	organoids	TI and SC	11	longitudinal samples	
223	paediatric	F	15 yrs	organoids	TI and SC	2		
223	paediatric	F	15 yrs	organoids	TI and SC	8	longitudinal samples	
224	paediatric	F	15 yrs	organoids	TI and SC	2 and 3		
224	paediatric	F	15 yrs	organoids	TI and SC	9	longitudinal samples	
225	paediatric	F	14 yrs	organoids	TI and SC	2		
229	paediatric	M	5 yrs	organoids	TI and SC	1		
242	paediatric	F	4 yrs	organoids	TI and SC	1		
F_1	foetal	M	GA 12 wk	purified	FPG and FDG	na		[1]
1320	foetal	M	GA 11 wk	purified	FPG and FDG	na		[1]
1327	foetal	M	GA 9 wk	purified	FPG and FDG	na		[1]
1324	foetal	F	GA 10 wk	purified	FPG and FDG	na		
1792	foetal	M	GA 10 wk	purified	FPG	na		
1690	foetal	F	GA 10 wk	purified	FPG	na		
1690	foetal	F	GA 10 wk	organoids	FPG and FDG	2		
1690	foetal	F	GA 10 wk	organoids	FPG and FDG	14	longitudinal samples	
1690	foetal	F	GA 10 wk	organoids	FPG and FDG	23	longitudinal samples	
1706	foetal	F	GA 9 wk	organoids	FPG and FDG	9		
1707	foetal	M	GA 9 wk	organoids	FPG and FDG	3		
1720	foetal	M	GA 10 wk	organoids	FPG and FDG	7		
1769	foetal	F	GA 11 wk	organoids	FPG and FDG	1 and 2		
1769	foetal	F	GA 11 wk	organoids	FPG and FDG	5	longitudinal samples	
1770	foetal	M	GA 10 wk	organoids	FPG and FDG	1		
1780	foetal	M	GA 11 wk	organoids	FPG and FDG	1 and 3		
39	paediatric	M	14 yrs	non-epithelial	TI, AC, SC	na		
WP1	paediatric	F	5-15 yrs	whole biopsy	SC	na		
WP2	paediatric	M	5-15 yrs	whole biopsy	SC	na		
WP3	paediatric	M	5-15 yrs	whole biopsy	SC	na		
WP4	paediatric	M	5-15 yrs	whole biopsy	SC	na		
WP5	paediatric	M	5-15 yrs	whole biopsy	SC	na		
WP6	paediatric	M	5-15 yrs	whole biopsy	SC	na		
200	paediatric	M	12 yrs	organoids	TI and SC	11	Diff and non-Diff Ctrl	
224	paediatric	F	15 yrs	organoids	TI and SC	11	Diff and non-Diff Ctr	
281	paediatric	F	2 yrs	organoids	GHR, REC, STO	3	GM and Ctrl	
281	paediatric	F	2 yrs	organoids	GHR, REC, STO	5	GM and Ctrl	
363	paediatric	M	4 yrs	organoids	TI and SC	4	Diff and non-Diff Ctr	
369	paediatric	M	9 yrs	organoids	TI and SC	3	Diff and non-Diff Ctr	
1769	foetal	F	GA 11 wk	organoids	FPG	8	TET1 KO	
1769	foetal	F	GA 11 wk	organoids	FPG	18	TET1 KO	
1769	foetal	F	GA 11 wk	organoids	FPG	12	WT Ctrl	
1769	foetal	F	GA 11 wk	organoids	FPG	21	WT Ctrl	
1888	foetal	F	GA 9 wk	organoids	FPG	2	Ctrl and AdC	
1888	foetal	F	GA 9 wk	organoids	FPG	6	Ctrl and AdC	
1889	foetal	M	GA 8 wk	organoids	FPG	2	Ctrl and AdC	
1889	foetal	M	GA 8 wk	organoids	FPG	6	Ctrl and AdC	

Table S1 continued

ID	Age group	Sex	Age	Sample Type	Gut Location	Passage	Treatments	Ref
A02	adult	F	60 yrs	organoids	SC	3		
A03	adult	M	35 yrs	organoids	SC	2		
A04	adult	F	26 yrs	organoids	TI and SC	4		
A06	adult	F	24 yrs	organoids	TI and SC	2		

TI= Terminal Ileum, AC=ascending colon; SC= Sigmoid Colon, GA= Gestational age, FPG= Foetal proximal gut, FDG= Foetal distal gut, yrs= years, GA= Gestational age, na= not applicable, Diff=Differentiation, Ctrl=Control, GM=Gastric medium, KO=Knockout, WT= Wildtype, STO=Stomach, GHR=Gastric Heterotopia in the rectum, REC=Rectum, AdC=Acadeoxycytidine, Ctrl=Control, Ref= Reference

Table S2. Patient information for the samples used for RNA sequencing

ID	Age group	Sex	Age	Sample Type	Gut Location	Passage	Reference
1	paediatric	M	13 yrs	purified	TI and SC	na	[2]
7	paediatric	F	14 yrs	purified	TI and SC	na	[2]
15	paediatric	F	15 yrs	purified	TI and SC	na	[2]
22	paediatric	F	14 yrs	purified	TI and SC	na	[2]
23	paediatric	M	14 yrs	purified	TI and SC	na	[2]
31	paediatric	M	9 yrs	purified	TI and SC	na	[2]
39	paediatric	M	14 yrs	purified	TI and SC	na	[2]
42	paediatric	M	15 yrs	purified	TI and SC	na	[2]
56	paediatric	F	13 yrs	purified	TI and SC	na	[2]
63	paediatric	M	6 yrs	purified	TI and SC	na	[2]
71	paediatric	M	9 yrs	purified	TI and SC	na	[2]
212	paediatric	M	11 yrs	organoids	TI and SC	6	
223	paediatric	F	15 yrs	organoids	TI and SC	2	
224	paediatric	F	15 yrs	organoids	TI and SC	2 and 3	
229	paediatric	M	5 yrs	organoids	TI and SC	1	
242	paediatric	F	4 yrs	organoids	TI and SC	1	
1792	foetal	M	GA nk	purified	FPG	na	
5	foetal	nk	GA 11 wk	purified	FPG	na	
8	foetal	nk	GA 9 wk	purified	FPG	na	
1324	foetal	F	GA 10 wk	purified	FPG	na	
1327	foetal	M	GA 9 wk	purified	FPG	na	
Pool1	foetal	nk	GA 9-11 wk	purified	FDG	na	
Pool2	foetal	nk	GA 9-11 wk	purified	FDG	na	
Pool3	foetal	nk	GA 9-11 wk	purified	FDG	na	
1690	foetal	F	GA 10 wk	organoids	FPG and FDG	2	
1720	foetal	M	GA 10 wk	organoids	FPG and FDG	7	
1769	foetal	F	GA 11 wk	organoids	FPG and FDG	1 and 2	
1770	foetal	M	GA 10 wk	organoids	FPG and FDG	1	
1780	foetal	M	GA 11 wk	organoids	FPG and FDG	1 and 3	

TI= Terminal Ileum, SC= Sigmoid Colon, GA= Gestational age, FPG= Foetal proximal gut, FDG= Foetal distal gut, wk=weeks, nk=not known, yrs=years, na= not applicable

Human intestinal epithelial organoid culture

The complete media composition for maintenance and differentiation of IEO as derived from [3,4] is shown in Tables S3 and S4. Media for newly seeded crypts was supplemented with 10 µM Y-27632 (Sigma). Conditioned media were kindly provided by Bon-Kyoung Koo (Wellcome Trust-MRC Stem Cell Institute, University of Cambridge, UK). Organoids were maintained with media change every 2 days and splitting by mechanical disruption every 7-10 days. Organoids were harvested by dissolving Matrigel with Cell Recovery Solution (Corning®) for 30 min on ice and 60 min at 4° C on a roller-shaker; washing with PBS and subsequent lysis in RLT Plus buffer with 1% β- Mercaptoethanol (Qiagen).

Table S3. Media composition for human intestinal organoids

**Basal Organoid medium = ADF+++**<br>Products from Gibco by Thermo Fisher Scientific	**Final concentration**
Advanced DMEM/F12 (ADF)	1x
GlutaMax	2 mM
HEPES buffer	10 mM
Penicillin/Streptomycin	0.5 U/ml
**Complete medium**	**Final concentration**
ADF+++ (see above)	27% (vol/vol)
Wnt3a- conditioned medium	50 % (vol/vol)
R-spo- conditioned medium	20 % (vol/vol)
Primocin (Invivogen, San Diego, CA, USA)	500 µg/mL
B-27® Supplement (Invitrogen, Carlsbad, CA, USA)	1x
Nicotinamide (Sigma, St. Louis, MO, USA)	10 mM
N-Acetylcysteine (Sigma, St. Louis, MO, USA)	1.25 mM
A-83-01 (Tocris, Bristol, UK)	500 nM
SB202190 (Sigma, St. Louis, MO, USA)	10 µM
Murine EGF (Invitrogen, Carlsbad, CA, USA)	50 ng/mL
Murine Noggin (Peprotech, Rocky Hill, NJ, USA)	100 ng/mL
Addition for foetal Organoids: PGE_2	2.5 µM

Table S4: Media composition for gastric and gastric heterotopic organoids

**Complete medium**	**Final concentration**
ADF+++ (see above)	25% (vol/vol)
Wnt3a- conditioned medium	50%
R-spo- conditioned medium	25%
B-27® Supplement (Invitrogen, Carlsbad, CA, USA)	1x
N-Acetylcysteine (Sigma, St. Louis, MO, USA)	1.25 mM
Nicotinamide (Sigma, St. Louis, MO, USA)	10 mM
Murine EGF (Invitrogen, Carlsbad, CA, USA)	20 ng/mL
Murine Noggin (Peprotech, Rocky Hill, NJ, USA)	150 ng/mL
FGF10 (Sigma, St. Louis, MO, USA)	150 ng/mL
Gastrin (Sigma, St. Louis, MO, USA)	10 nM
A-83-01 (Tocris, Bristol, UK)	1 µM
SB202190 (Sigma, St. Louis, MO, USA)	2 µM

Table S5: Media composition for in vitro differentiation of IEO

Complete medium	Final concentration
ADF+++ (see above)	77% (vol/vol)
R-spo- conditioned medium	20 % (vol/vol)
Primocin (Invivogen, San Diego, CA, USA)	500 µg/mL
B-27® Supplement (Invitrogen, Carlsbad, CA, USA)	1x
N-Acetylcysteine (Sigma, St. Louis, MO, USA)	1.25 mM
A-83-01 (Tocris, Bristol, UK)	500 nM
Murine EGF (Invitrogen, Carlsbad, CA, USA)	50 ng/mL
Murine Noggin (Peprotech, Rocky Hill, NJ, USA)	100 ng/mL

Table S6. Primers used for PCR and pyrosequencing

Gene	Forward primer [5'-3']	Reverse primer [5'-3']	Sequencing primer [5'-3']
GATA2	GTTGGGTAGAAAGGA GAGGGATAAAAGA	[Btn]-AATAATAACAAAAATCACCCAACTCA ACCT	AAAAGAGGAGAGGGG
HHEX	TTTGTAAGAAGGGGTT TTAGAGTAG	[Btn]-TAACCTCCTAACCTACCTTTATCAT	AGGGGTTTTAGAGTA GA
IL6R	GGTTGGGGTAGAGGG GTTTAGTTA	[Btn]-CCCCACTCACAAACAACATTACT	GTTTTTTTTAAGGGAG GT
SATB2AS1	TTTTTAGTTTAGGATG TTTTGGGATAT	[Btn]-AAAACCCCAATTAACTATCTATTATAT CAA	GGGATATTAGGATTTA GGAGAGA
TET1	AGATTTTGGAGAGAG AATAGGATTAGGTA	[Btn]-ATACTCCAAATAACTATTACCCTTACT	GGAGAGAGAATAGGA TTAGGTATA
TLR4	ATGATTAATTGGGATA AAAGTTAATTAGTT	[Btn]-CTACTTCTATAAACAACAATAACCCTA TA	AGAGAAGATATTAGTG TTTTAG

Btn=5'-biotinylation

Table S7. Gene expression primers used for real-time PCR

Gene	Forward primer [5'-3']	Reverse Primer [5'-3']
ACTA2	AAAAGACAGCTACGTGGGTGA	GCCATGTTCTATCGGGTACTTC
ALPI	CATGGACCGCTTCCCATA	GGCACCTGTCTGTCCACAT
CD45	ACCACAAGTTTACTAACGCAAGT	TTTGAGGGGGATTCCAGGTAAT
CFTR	AGTGGAGGAAAGCCTTTGGAGT	ACAGATCTGAGCCCAACCTCA
CLDN15	GGCTTCTTCATGGCAACTGT	GGGAACTCCCAGCAGTTGTA
FABP6	ACTACTCCGGGGGCCACACCAT	GTCTCTTGCTCACGCGCTCATAGG
GAPDH	AGGTCGGAGTCAACGGATTT	TGGAAGATGGTGATGGGATTT
Ki67	GAGAGTAACGCGGAGTGTCA	CCCAGTTCGATTTTTCTGTCA
LGR5	CTCCCAGGTCTGGTGTGTTG	GAGGTCTAGGTAGGAGGTGAAG
LRIG	GGACTTGCCGAACCTACAGG	GCTGCGAATCTTGTTGTGCTG
LYZ	TCAATAGCCGCTACTGGTGTA	ATCACGGACAACCCTCTTTGC
MUC12	CCAGTTCAAGCGACCCTTTTA	CGCTGTGGGATACTGTTGATT
MUC17	ATAGGGCCACCGGAGACTAT	TGCCAAGACGTAGCTTTGTG
MUC2	GATTCGAAGTGAAGAGCAAG	CACTTGGAGGAATAAACTGG
MUC4	CGTTCTGGGACGATGCTGAC	GATGGCTTGGTAGGTGTTGCT
MUC5B	GTGCCCTTGCACTGTCCTAACG	ACAATGCACATCCTGGCTCCAG
NR1H4	CCCCAAGTTCAACCACAGAT	CTTTGATCCTCCCTGCTGAC
PECAM1	AACAGTGTTGACATGAAGAGCC	TGTAAAACAGCACGTCATCCTT
SATB2	CTTTGCAAGAGTGGCATTCA	GTTGTCGGTGTCGAGGTTTT
SI	TCATGTATGGGGGAAAGGAA	TGTGTGTGGTCAGATCAATACG
SLC5A1	ATCTATATTAAGGCTGGGGTG	TCTTGGTGAAAATGTAGAGC
SOX9	CTCTGGAGACTTCTGAACGAGAG	CCTTGAAGATGGCGTTGGGG
TFF3	GGAGTGCCTTGGTGTTTCAA	CCCACGACGCAGCAGAAA
VIL1	CTGAGCGCCCAAGTCAAAG	AGCAGTCACCATCGAAGAAGC
VIM	GACGCCATCAACACCGAGTT	CTTTGTCGTTGGTTAGCTGGT

RNA sequencing

Libraries for RNA sequencing were prepared with Illumina TruSeq protocol (Illumina, Cambridge, UK). RNA sequencing was performed using paired-end reads on the Illumina HiSeq 2500 platform (Illumina, Cambridge, UK) at the Institute of Clinical Molecular Biology at the University of Kiel (Kiel, Germany) and the Wellcome Trust Sanger Institute (Hinxton, UK).

Bioinformatic analysis

DNA methylation analysis

Pre-processing included filtering of probes for significant detection p-value (<e10^{-5}), functional normalization, exclusion of probes located on X and Y chromosomes or probes co-locating with SNPs was performed using the Bioconductor package *minfi* [5]. Batch correction was achieved using the combat function in the package *sva* [6]. Beta and M values were called using *minfi* [5]. Differential methylation analysis was performed on M-values using *limma* [7] for differentially methylated positions (DMPs) (adjusted p <0.01) and *DMRcate* [8] for differentially methylated regions (DMRs). Heatmaps based on M-values were created using *heatmap3* [9].

RNA sequencing analysis

Pre-processing of sequencing data included removal of low quality reads using *fastq_illumina_filter* and trimming of adapters using *cutadapt* [10]. Filtered reads were subsequently mapped to the human genome using the GRCh37 reference with *tophat2* [11], *bowtie* [12] and *samtools* [13]. Raw read alignments were counted using *htseq-count* [14]. Batch correction was performed using *RUVseq* based on expression of housekeeping genes as control genes by calling the *ruvg* function [15]. Differential gene expression analysis was performed on normalized counts using *DESeq2* [16] with a cut-off of adjusted $p<0.01$ and Log_2FoldChange >1.5 or <-1.5. Heatmaps were created on rlog-transformed read counts using *heatmap3* [9] Gene ontology analysis was performed using *GOseq* [17].

Immunofluorescence and imaging

Organoids in Matrigel were fixed with 4% formaldehyde (Sigma) for 20 min at room temperature, washed and blocked for 1 hour in blocking buffer [10% goat serum (New England Biolabs) +1% BSA +0.5% Triton-X (both Sigma)]. Primary antibody [Anti-FABP6 (1:500, HPA012601, Atlas Antibodies), anti-MUC5B (1:100, HPA008246, Atlas

Antibodies), or anti-EpCAM (1:200, ab20160, Abcam)] was applied in blocking buffer overnight at 4°C. After washing, goat anti-rabbit IgG (Life Technologies) or goat-anti mouse IgG (abcam) was applied 1:1000 for 1h at room temperature with added nuclear dye DAPI (1µg/ml) (Sigma). Organoids were washed 5 times with PBS+0.05% Tween-20 (Sigma) and imaged. Fluorescent and brightfield images were obtained using an EVOS FL system (Life Technologies).

Vector construction and genome editing

The targeting vector was generated by PCR-amplification of homology arms from human genomic DNA using Phusion polymerase kit (New England Biolabs) (Table S8) and assembly into pUC118-FLIP-Puro vector backbone (Addgene #84538,[18]) via Golden Gate cloning reaction. Recombined plasmids were tested for the correct insert by restriction digest and Sanger-sequencing at the Department of Biochemistry, University of Cambridge. For the sgRNA vector, the TET1-specific gRNA was amplified from the generic gRNA vector template by reverse PCR using Phusion HF polymerase (New England Biolabs) (Table S9).

Table S8: Primer sequences for amplification of homology arms

**Forward Primer [5'-3'] for 5'Arm**	**Reverse Primer [5'-3'] for 5'Arm**
TGTGCACCACCACACTCAGCTAA	CACACAAGGTTTTGGTCTGAAATAACAACA
**Forward Primer [5'-3'] for 3'Arm**	**Reverse Primer [5'-3'] for 3'Arm**
GGGAGACAGCTGACACTTGGTATAGTGA	CACCACACCAGGCCTACAATACTTCTAA

Table S9: Primer sequences for PCR amplification of vector

**Forward primer [5'-3']**	**Specific reverse primer [5'-3']**
[Phos]-GTTTTAGAGCTAGAAATAGCAAGTTAAAATAAGG	TGCATGGCGGGATCGAGACACGGTGTTTCGTCCTTTCCACAAGAT

Phos= 5'phosphorylation.

Human IEOs were cultured in conditioned-medium-free medium (Table S10) and electroporated following the protocol from Fujii et al [19]. Puromycin (2µg/ml) was used for selection on day 7 after electroporation.

Genotyping of individual organoid clones was performed by PCR reaction targeting each of the junctions of the integrated cassette (Table S11). Sequencing of the genomic WT allele (TET1 exon2, Table S11), as well as the TET1 transcript from cDNA (spanning exon1 - exon2) was performed by Sanger sequencing by Source Bioscience (Cambridge, UK) (Table S12).

Table S10: Medium used for organoids before and after electroporation

Reagent	Final concentration
AdF+++=<br>Advanced DMEM/F12 +1%GlutaMax+ 1 mM HEPES<br>(all Gibco by Life Technologies)	97.5 % (vol/vol)
B 27 supplement (Invitrogen)	1x
Nicotinamide (Sigma)	10 mM
N-Acetylcytsteine (Sigma)	1.25 mM
EGF (Invitrogen)	50 ng/ml
Noggin (Peprotech)	100 ng/ml
A83-01 (Tocris)	500 nM
SB202190 (Sigma)	10 µM
15-Leu Gastrin I (Sigma)	10 nM
CHIR99021 (Cheyman Chemical)	5 µM
**Optional depending on time point**	
DMSO (Sigma)	1.25 % (vol/vol)
Y-27632 (Sigma)	10 µM

Table S11: Primer sequences for amplification of cassette integration site and for amplification and sequencing of WT allele in exon2

Target	Forward primer [5'-3']	Reverse primer [5'-3']
3'-arm junction	TGAGACGTGCTACTTCCATTTGTCA	TGTAAACCACCATGCCCAACCACAA
5'arm junction	GCCCAGCCAGTACCGGATAGTTTTT	CTTCTAGTTGCCAGCCATCTGTTGT
EXON2	GGAAGATTGCTTGAGCCTGGGTAGG	CCCATTGCAGGTTAAGGACTCTGGG

Table S12: Primer sequences for amplification and sequencing of TET1 mRNA spanning exon1-exon2 junction

Target	Forward primer [5'-3']	Reverse primer [5'-3']
TET1 mRNA	CGAGTTGGAAAGTTTGCCCG	GCACGGGTGGTTTAGGTTCT

References

1 Kraiczy J, Nayak K, Ross A, *et al.* Assessing DNA methylation in the developing human intestinal epithelium: potential link to inflammatory bowel disease. *Mucosal Immunol* 2016;**9**:647–58. doi:10.1038/mi.2015.88

2 Howell K, Kraiczy J, Nayak K, *et al.* DNA Methylation and Transcription Patterns in Intestinal Epithelial Cells From Pediatric Patients With Inflammatory Bowel Diseases Differentiate Disease Subtypes and Associate With Outcome. *Gastroenterology* 2017;**In press.** doi:10.1053/j.gastro.2017.10.007

3 Sato T, Stange DE, Ferrante M, *et al.* Long-term expansion of epithelial organoids from human colon, adenoma, adenocarcinoma, and Barrett's epithelium. *Gastroenterology* 2011;**141**:1762–72. doi:10.1053/j.gastro.2011.07.050

4 Fordham RP, Yui S, Hannan NRF, *et al.* Transplantation of expanded fetal intestinal progenitors contributes to colon regeneration after injury. *Cell Stem Cell* 2013;**13**:734–44. doi:10.1016/j.stem.2013.09.015

5 Aryee MJ, Jaffe AE, Corrada-Bravo H, *et al.* Minfi: a flexible and comprehensive Bioconductor package for the analysis of Infinium DNA methylation microarrays. *Bioinformatics* 2014;**30**:1363–9. doi:10.1093/bioinformatics/btu049

6 Leek JT, Johnson WE, Parker HS, Fertig EJ JA and SJ. sva: Surrogate

Variable Analysis. *R Packag version 3180*

7 Ritchie ME, Phipson B, Wu D, *et al.* limma powers differential expression analyses for RNA-sequencing and microarray studies. *Nucleic Acids Res* 2015;**43**:e47. doi:10.1093/nar/gkv007

8 Peters TJ, Buckley MJ, Statham AL, *et al.* De novo identification of differentially methylated regions in the human genome. *Epigenetics Chromatin* 2015;**8**:6. doi:10.1186/1756-8935-8-6

9 Zhao S, Guo Y, Sheng Q, *et al.* heatmap3: An Improved Heatmap Package. 2015.https://cran.r-project.org/web/packages/heatmap3/index.html

10 Martin M. Cutadapt removes adapter sequences from high-throughput sequencing reads. *EMBnet.journal* 2011;**17**:10. doi:10.14806/ej.17.1.200

11 Kim D, Pertea G, Trapnell C, *et al.* TopHat2: accurate alignment of transcriptomes in the presence of insertions, deletions and gene fusions. *Genome Biol* 2013;**14**:R36. doi:10.1186/gb-2013-14-4-r36

12 Langmead B. Aligning short sequencing reads with Bowtie. *Curr Protoc Bioinformatics* 2010;**Chapter 11**:Unit 11.7. doi:10.1002/0471250953.bi1107s32

13 Li H, Handsaker B, Wysoker A, *et al.* The Sequence Alignment/Map format and SAMtools. *Bioinformatics* 2009;**25**:2078–9. doi:10.1093/bioinformatics/btp352

14 Anders S, Pyl PT, Huber W. HTSeq - A Python framework to work with high-throughput sequencing data. *Bioinformatics* 2014;**31**:166–9. doi:10.1093/bioinformatics/btu638

15 Risso D, Ngai J, Speed TP, *et al.* Normalization of RNA-seq data using factor analysis of control genes or samples. *Nat Biotechnol* 2014;**32**:896–902. doi:10.1038/nbt.2931

16 Love MI, Huber W, Anders S. Moderated estimation of fold change and dispersion for RNA-seq data with DESeq2. *Genome Biol* 2014;**15**:550. doi:10.1186/s13059-014-0550-8

17 Young MD, Wakefield MJ, Smyth GK, *et al.* Gene ontology analysis for RNA-seq: accounting for selection bias. *Genome Biol* 2010;**11**:R14. doi:10.1186/gb-2010-11-2-r14

18 Andersson-Rolf A, Mustata RC, Merenda A, *et al.* One-step generation of conditional and reversible gene knockouts. *Nat Methods* 2017;**14**:287–9. doi:10.1038/nmeth.4156

19 Fujii M, Matano M, Nanki K, *et al.* Efficient genetic engineering of human intestinal organoids using electroporation. *Nat Protoc* 2015;**10**:1474–85. doi:10.1038/nprot.2015.088
