# Supplementary material for: DNA methylation defines regional identity of human intestinal epithelial organoids and undergoes dynamic changes during development
Source: Gut. 2017 Nov 15;68(1):49–61. doi: 10.1136/gutjnl-2017-314817 (PMC6839835; doi:10.1136/gutjnl-2017-314817)
Supplement: Supplementary file 2 [file gutjnl-2017-314817supp002.pdf]

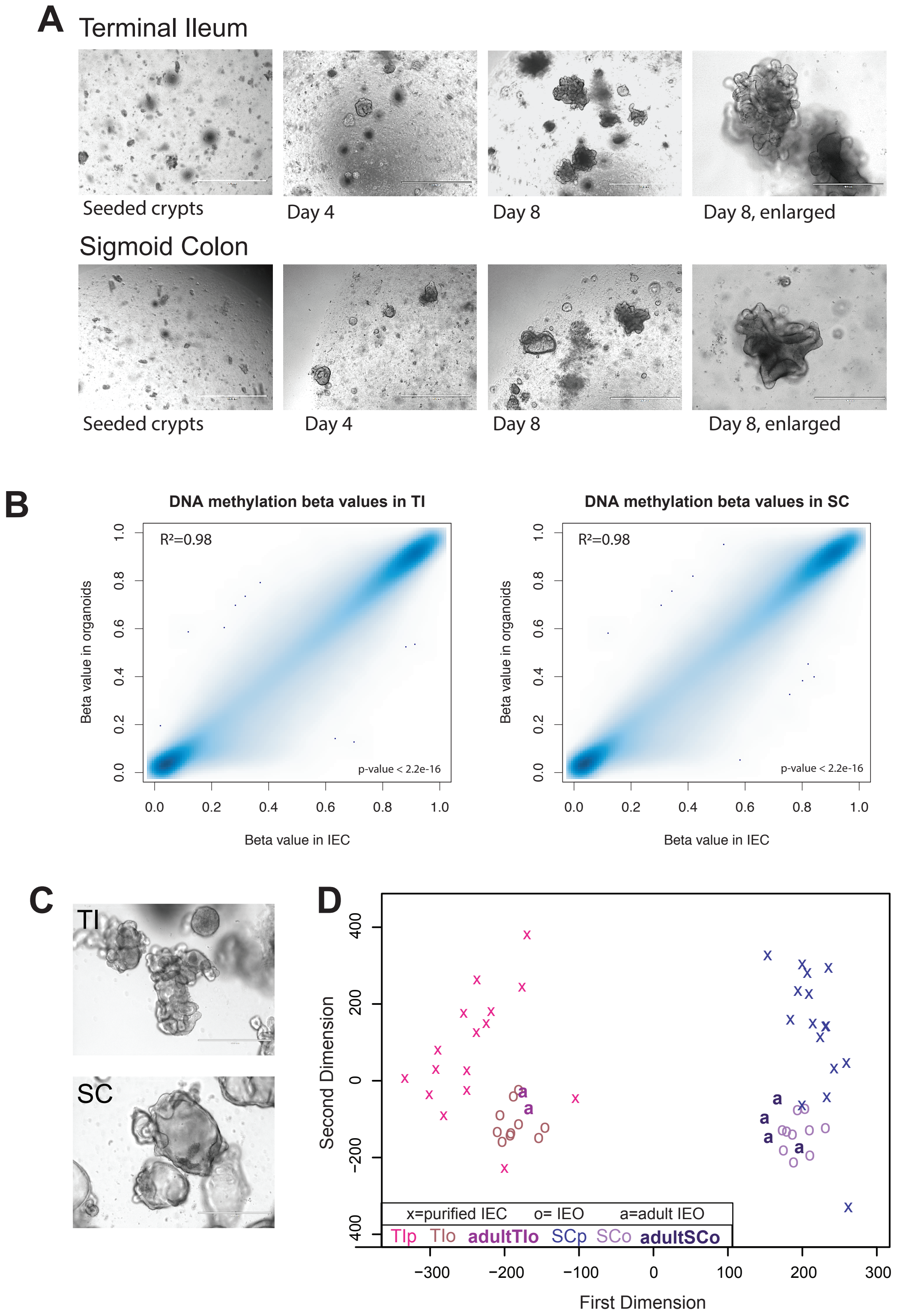

Supplementary Figure S1. Extended characterization of human intestinal epithelial organoids (IEO) derived from ileum and colon. (A) Light microscopy images of human intestinal organoids grown from terminal ileum (upper panel) and sigmoid colon (lower panel) over 8 days. Scale bar represents 1000 µm in pictures 1-3 and 400 µm in picture 4 (left to right). (B) Correlation of all DNA methylation values (415,550 CpGs) in purified IEC (x-axis) and IEO from the same segment (y-axis). Shown are average beta values in a matched sample set of n=7 individuals; Pearson's correlation r=0.9915 (TI) and r=0.9916 (SC), R^2=0.98, p <2.2 e^{-16}. (C) Representative brightfield images of IEO derived from TI and SC from a healthy adult. (D) MDS plot of the genome wide DNA methylation profiles of adult IEO (TI and SC) in the context of paediatric samples, both purified IEC and IEO (see also Figure 1B). Adult and paediatric organoids cluster closely together with the respective gut segment. SCp= Sigmoid colon purified, SCo=Sigmoid colon organoids, TIp= Terminal ileum purified, TIo= Terminal ileum organoids.

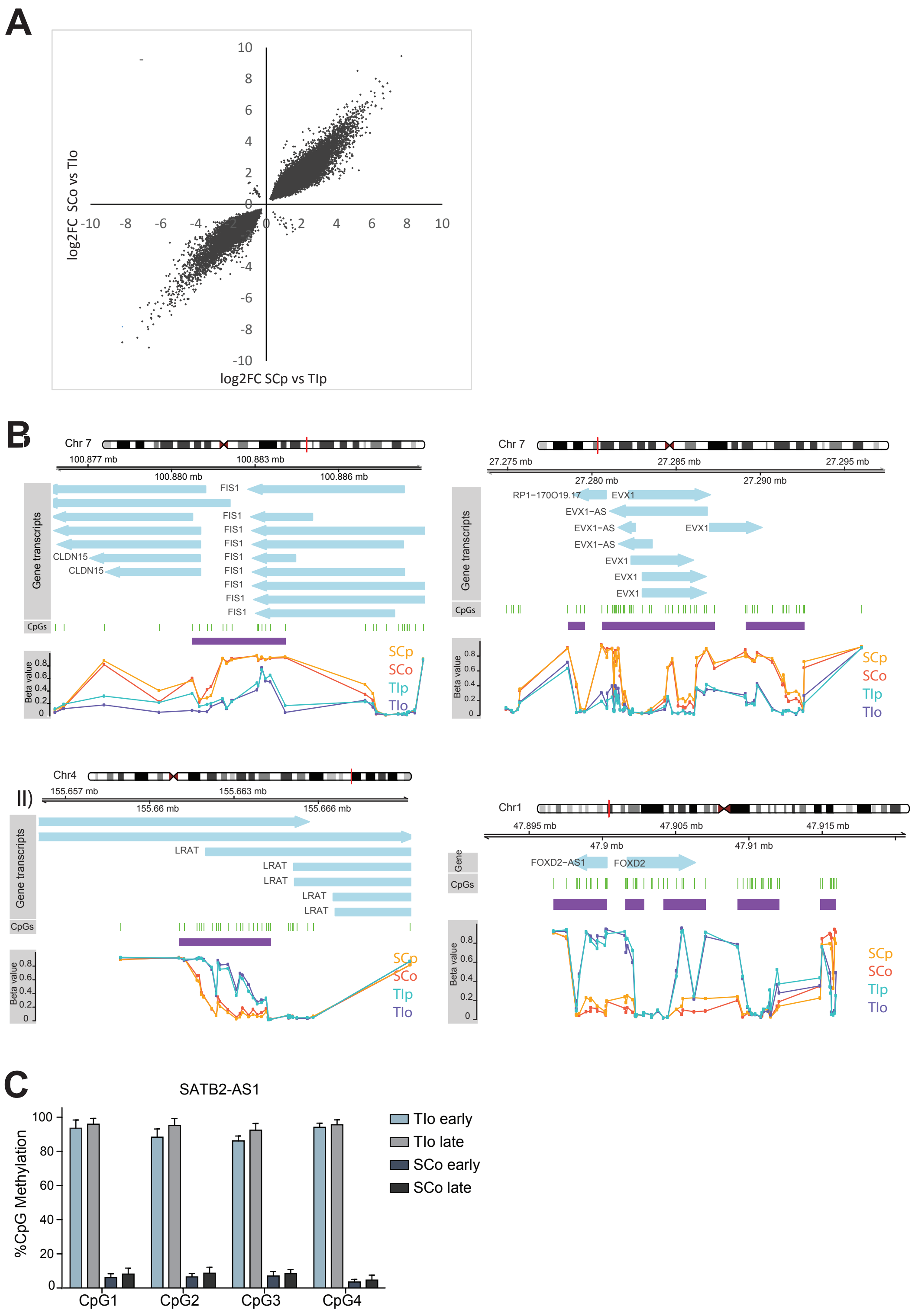

Supplementary Figure S2. DNA methylation differences in epithelial cells from TI and SC. (A) Scatterplot of log-fold change values of DMPs (SC vs TI, adj p <0.01, 23,450 DMPS total) that are overlapping in both IEC and IEO (see also Figure 1C). (B) Representative examples of gut segment -specific differentially methylated regions (DMRs) displaying highly conserved methylation profiles in intestinal organoids derived from the same gut segment. I) Hypermethylation in the colon in DMRs located in the genes for Fission, mitochondrial 1 (FIS1) and the tight junction protein Claudin 15 (CLDN15) (both left), as well as the transcription factor Even-Skipped Homeobox 1 (EVX1) (upper right). II) DMRs with hypermethylation in the ileum in the genes for Lecithin Retinol Acyltransferase (LRAT) (left) and Forkhead Box D2 (FOXD2) (right). Sample data is shown as average beta value per group along the genomic coordinates in hg19 and gene transcript models. n= 16 (IEC) and 7 (IEO) per gut segment. (C) Pyrosequencing data showing stable DNA methylation levels in the gene for SATB2-AS1 comparing early (Passage 1-5) and late (Passage 10-17) TIo and SCo. Data is represented as mean+SD of n=4-5 per group.

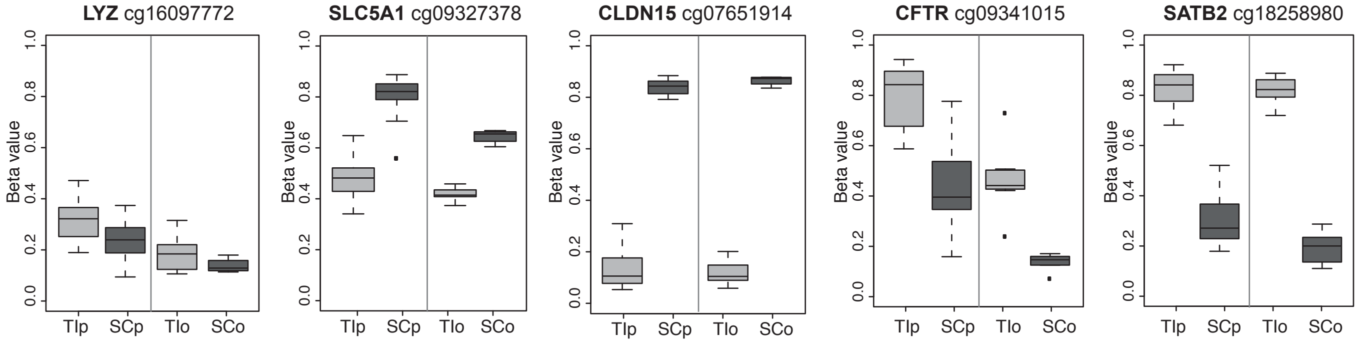

Supplementary Figure S3: DNA methylation beta values of CpGs in the genes shown in Figure 2D and E. Box-Whisker plots derived from genome-wide profiles of samples of n=16 (TIp and SCp) and 7 (TIo and SCo). Header refers to gene symbol and Illumina CpG identifier on the array. LYZ=Lysozyme, SLC5A1= Solute Carrier Family 5 Member 1, CFTR= Cystic-Fibrosis-Transmembrane Conductance Regulator.

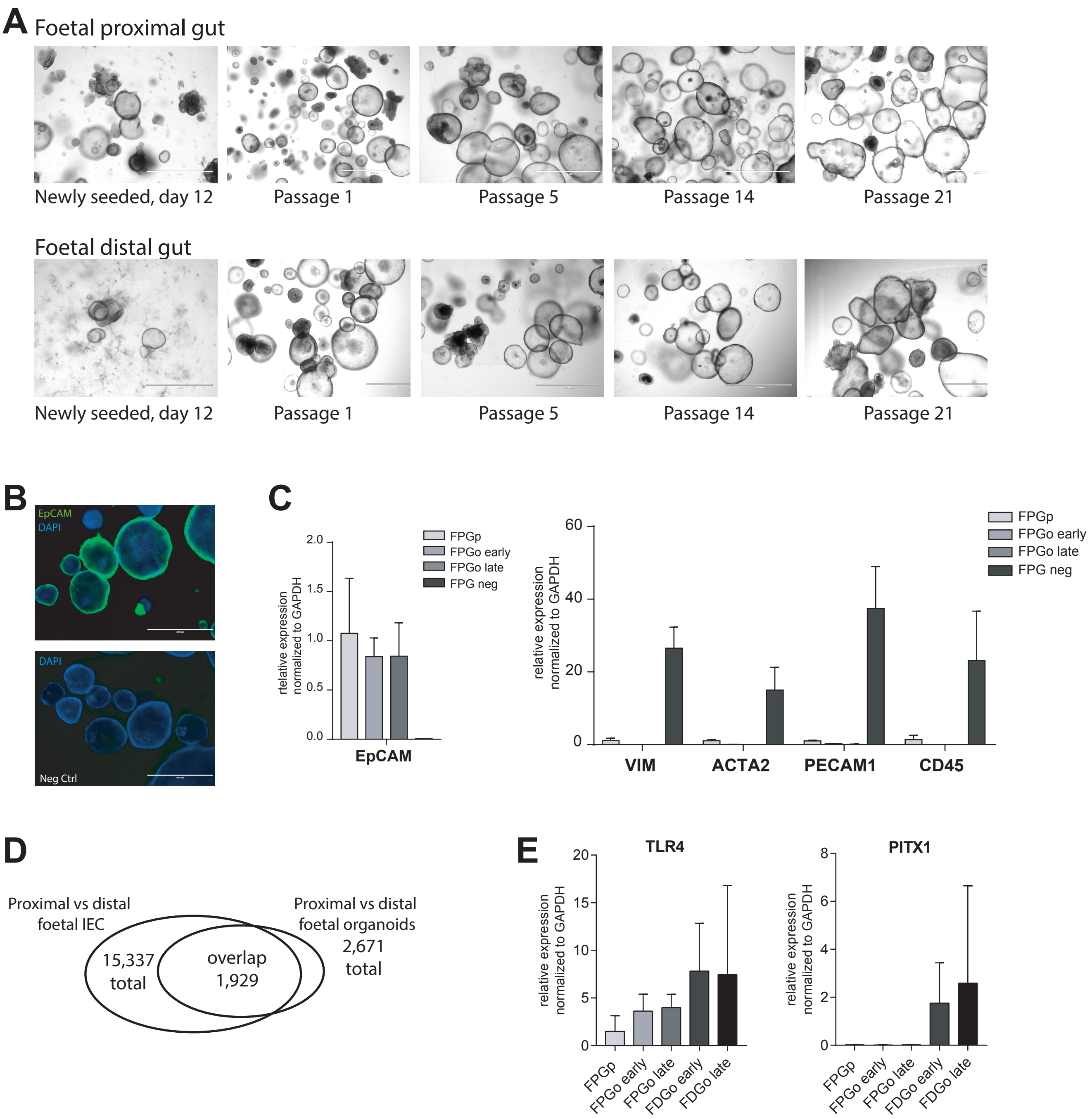

Supplementary Figure S4. Extended characterization of human foetal IEO. (A) Representative brightfield images of human foetal IEO over 21 passages. Upper Panel shows organoids derived from foetal proximal gut (i.e. small intestine), lower panel shows organoids derived from foetal distal gut (i.e. large intestine) (B) Immunofluorescent staining for epithelial marker EpCAM (green) and negative staining control (no primary antibody) in foetal IEO. Scale bar represents 400µm. Blue=cell nuclei (DAPI). (C) Confirmation of epithelial cell purity in foetal organoid cultures. Gene expression of EpCAM (left) and non-epithelial marker (right) measured by qPCR in foetal purified IEC, early FPGo (passages 1-2), late FPGo (passages 10-14) as well as non-epithelial cells, i.e. sorted EpCAM-negative fraction from the purification protocol (FPGneg). Mean+SD of n=2 (FPGp) and 4 (other groups), all groups are plotted. VIM=Vimentin (mesenchymal cells), ACTA2= Alpha smooth muscle actin (muscle cells), PECAM1= Platelet And Endothelial Cell Adhesion Molecule 1 (Endothelial cells), CD45= Cluster of Differentiation 45 (leukocytes). (D) Venn diagram of significant DMPs ($p<0.01$) that overlap between the comparisons “foetal proximal vs distal IEC” and “foetal proximal vs distal IEO”. (E) qPCR gene expression of Toll-like receptor 4 (TLR4) and Paired Like Homeodomain 1 (PITX1) in FPGp, early (passages 1-3) and late (passages 10-16) foetal IEO. Data expressed as mean+SD of n=4-6 per group.

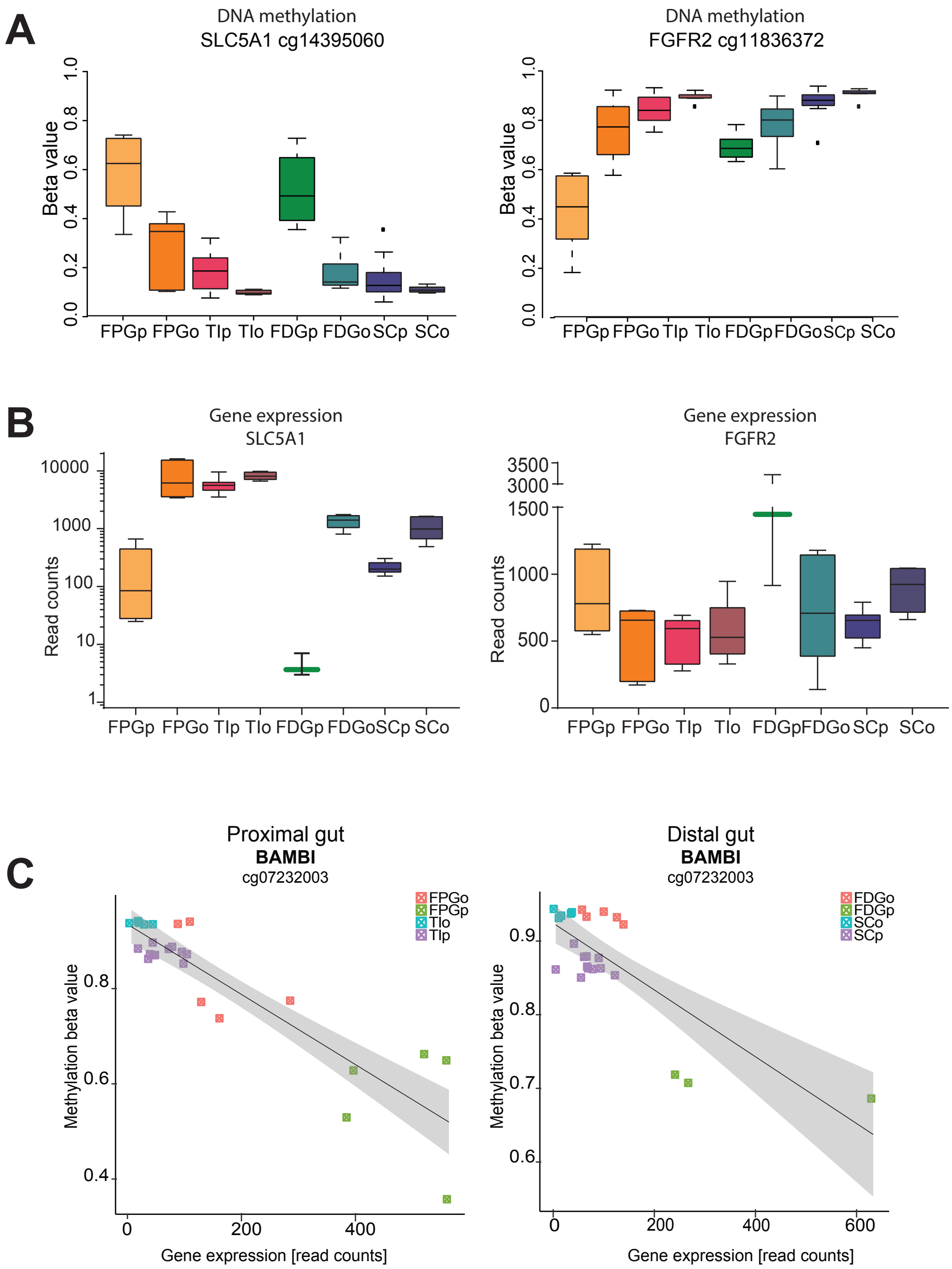

Supplementary Figure S5. Correlation between DNA methylation and gene expression in human foetal IEO. (A) DNA methylation beta values of CpG positions within the genes for the major glucose transporter Solute Carrier Family 5 Member 1 (*SLC5A1*), left, and Fibroblast Growth Factor Receptor 2 (*FGFR2*), right. Foetal organoids show either loss of methylation (*SLC5A1*) or gain in methylation (*FGFR2*) compared to the respective purified foetal epithelium and change in the direction of the corresponding paediatric gut segment. Boxplot of n= 5-16 per group. (B) Corresponding gene expression counts for the genes shown in A, *SLC5A1* and *FGFR2*, illustrating inverse changes in gene expression compared to the respective DNA methylation profile. Boxplot of n= 3-11 per group. (C) Example correlation plot of DNA methylation and gene expression: Gene expression values [RNA seq read counts] and methylation values for one CpG in BMP And Activin Membrane Bound Inhibitor (*BAMBI*) in the different sample groups. Each point represents one sample, trend indicated as line.

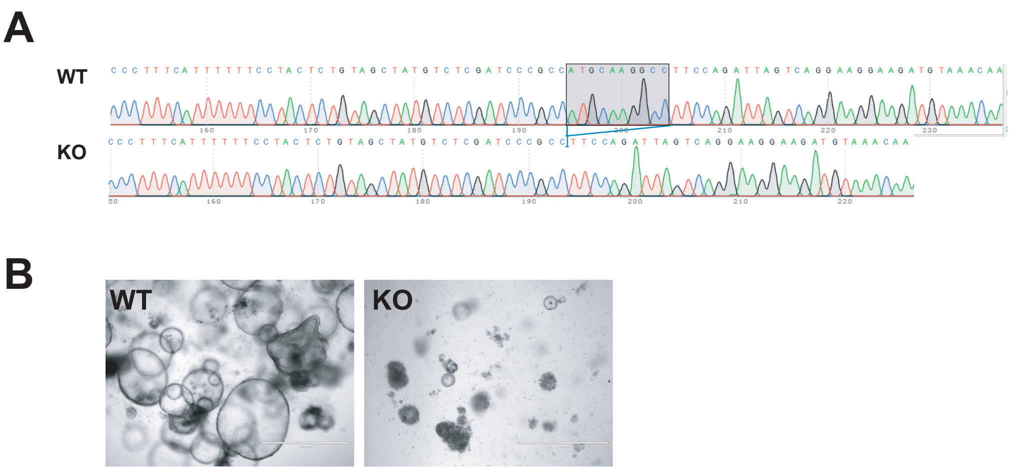

Supplementary Figure S6. Alterations in human FPGo with TET1-disruption. (A) RNA –Sanger sequencing traces of TET1 transcript of WT and TET1-KO FPGo. Box marks the 10 bases that are missing in KO, leading to altered mRNA with frameshift. (B) Brightfield images of FPGo in long-term culture at passage 29 (WT) and 24 (KO)

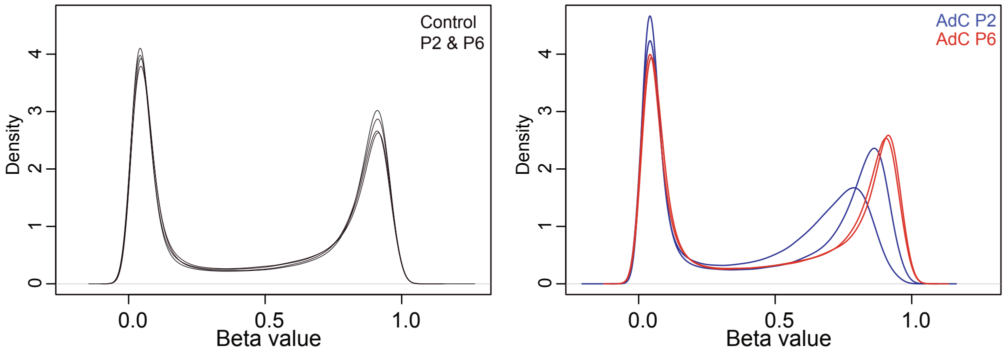

Supplementary Figure S7. Loss and regain of DNA methylation after DNA methyl-transferase inhibitor treatment. Density plot showing the distribution of beta values across all genome-wide probes in foetal proximal gut organoids (FPGo). Left panel shows control samples, right panel shows samples one passage (AdC P2) and five passages (AdC P6) after initial Aza-deoxycytidine (AdC) treatment at P1. Each line represents one sample. AdC treatment causes global demethylation as indicated by a decrease in the peak over the beta value of 1.0 and an increase in the peak over the beta value of 0. Five passages after treatment, organoids have regained DNA methylation to levels similar to non-treated controls.

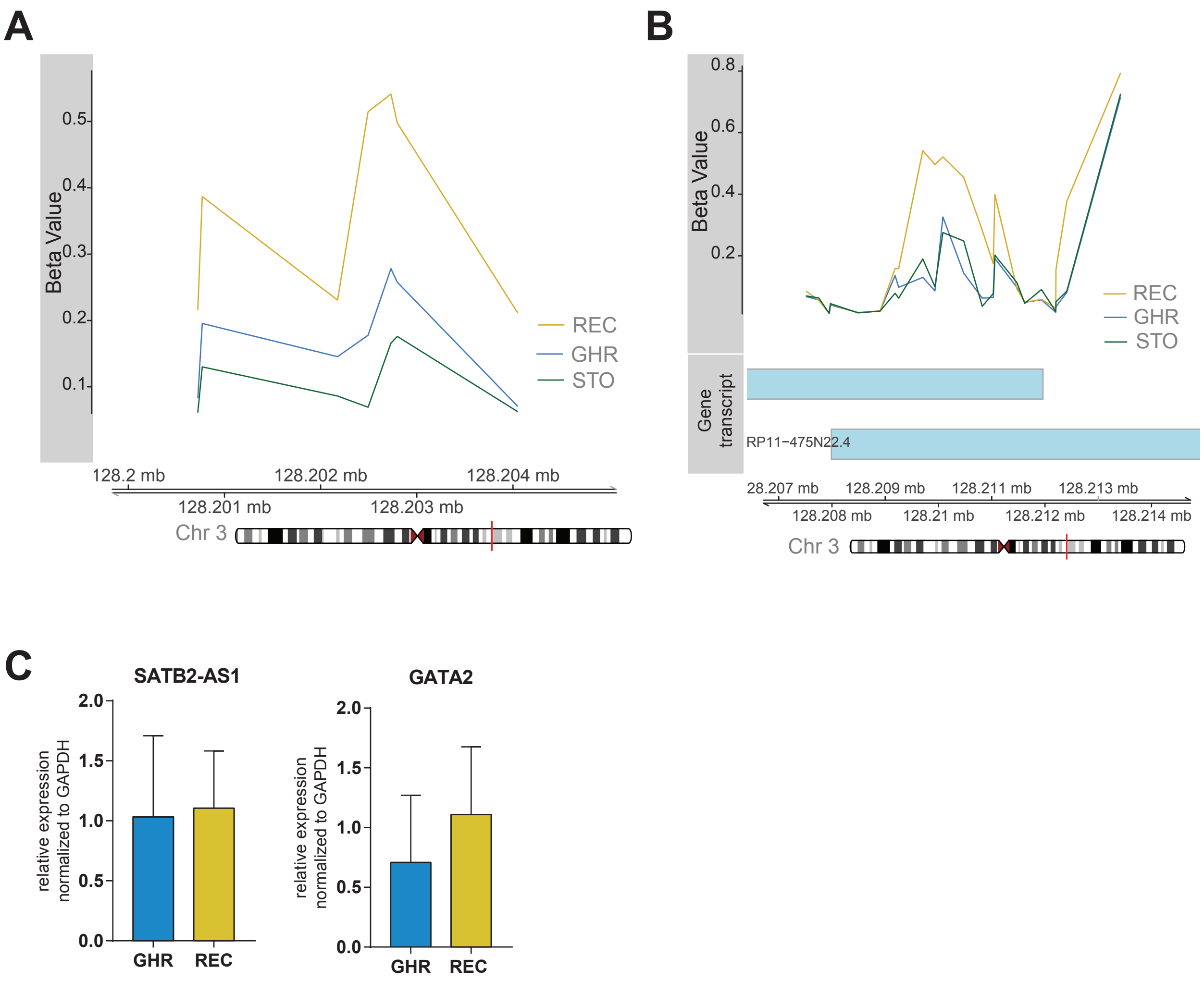

Supplementary FigureS8. Differentially methylated regions between rectal and gastric heterotopic IEO. Beta values along the genomic coordinates in hg19 and gene transcript models (A) downstream of the gene for the transcription factor GATA-binding protein 2 (*GATA2*) and (B) in the lncRNA locus *RP11-475N22.4* located upstream of GATA2. Sample data is shown as average beta value per group (n=3 for rectal and heterotopic IEO, n=2=for stomach organoids, derived from the same individual donor). (C) Gene expression of SATB2-AS1 and GATA2 in gastric heterotopic organoids (GHR) and rectal (REC) organoids. Data expressed as mean+SD, n=4 per group.
